# Genomic regions occupied by both RARα and VDR are involved in the convergence and cooperation of retinoid and vitamin D signaling pathways

**DOI:** 10.1093/nar/gkaf230

**Published:** 2025-04-01

**Authors:** Hamidreza Mianesaz, Loránd Göczi, Gergely Nagy, Szilárd Póliska, Lina Fadel, Dóra Bojcsuk, András Penyige, Krisztina Szirák, Farah AlHaman, László Nagy, György Vámosi, Lajos Széles

**Affiliations:** Department of Human Genetics, Faculty of Medicine, University of Debrecen, Debrecen H-4032, Hungary; Department of Human Genetics, Faculty of Medicine, University of Debrecen, Debrecen H-4032, Hungary; Department of Biochemistry and Molecular Biology, Doctoral School of Molecular Cell and Immune Biology, Faculty of Medicine, University of Debrecen, Debrecen H-4032, Hungary; Department of Biochemistry and Molecular Biology, Doctoral School of Molecular Cell and Immune Biology, Faculty of Medicine, University of Debrecen, Debrecen H-4032, Hungary; Institute for Diabetes and Endocrinology IDE, Helmholtz Munich, 85764 Neuherberg, Germany; Department of Biochemistry and Molecular Biology, Doctoral School of Molecular Cell and Immune Biology, Faculty of Medicine, University of Debrecen, Debrecen H-4032, Hungary; Department of Human Genetics, Faculty of Medicine, University of Debrecen, Debrecen H-4032, Hungary; Department of Human Genetics, Faculty of Medicine, University of Debrecen, Debrecen H-4032, Hungary; Department of Human Genetics, Faculty of Medicine, University of Debrecen, Debrecen H-4032, Hungary; Department of Biochemistry and Molecular Biology, Doctoral School of Molecular Cell and Immune Biology, Faculty of Medicine, University of Debrecen, Debrecen H-4032, Hungary; Department of Medicine and Biological Chemistry, Johns Hopkins University School of Medicine, Institute for Fundamental Biomedical Research, Johns Hopkins All Children's Hospital, Saint Petersburg, Florida 33701, United States; Department of Biophysics and Cell Biology, Faculty of Medicine, Doctoral School of Molecular Medicine, University of Debrecen, Debrecen H-4032, Hungary; Department of Human Genetics, Faculty of Medicine, University of Debrecen, Debrecen H-4032, Hungary

## Abstract

Retinoic acid receptors (RARs) and the vitamin D receptor (VDR) regulate distinct but overlapping gene sets in multiple cell types. The abundance and characteristics of regulatory regions, occupied by both RARs and VDR are largely unexplored. We used global approaches (ChIP-seq, RNA-seq, and ATAC-seq) and bioinformatics tools to map and characterize common binding regions of RARα and VDR in differentiated human THP-1 cells. We found that the cistromes of ligand-activated RARα and VDR largely overlapped, and their agonists (AM580 and calcitriol) co-regulated several genes, often cooperatively. Common binding regions were frequently (but not exclusively) annotated with co-regulated genes and exhibited increased MED1 occupancy upon ligand stimulation, suggesting their involvement in gene regulation. Chromatin accessibility was typically higher in the common regions than in regions occupied exclusively by RARα or VDR. DNA response elements for RARα (DR1/2/5) and VDR (DR3) were enriched in the common regions, albeit the co-occurrence of the two types of canonical motifs was low (8.4%), suggesting that “degenerate” DR1/2/5 and DR3 motifs or other sequences could mediate the binding. In summary, common binding regions of RARα and VDR are at the crossroads of the retinoid and vitamin D pathways, playing important roles in their convergence and cooperation.

## Graphical Abstract

**Graphical Abstract:**
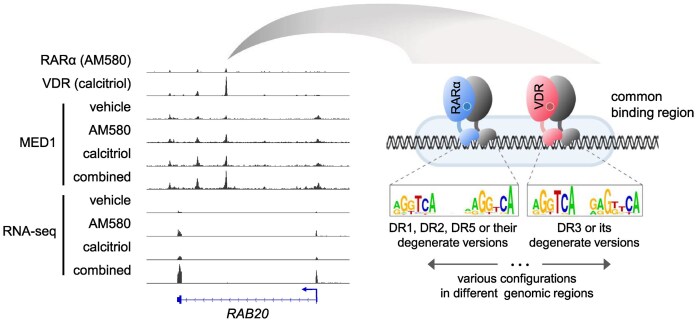

## Introduction

Retinoic acid receptors (RARs) and the vitamin D receptor (VDR) play essential roles in mediating the effects of retinoids (vitamin A and related compounds) and vitamin D, respectively [1–4]. Retinoids and vitamin D are essential for normal human development and health. Vitamin A deficiency increases susceptibility to severe infections, correctable blindness, skin diseases, and abnormal embryonic development [4]. Vitamin D deficiency is associated with hypocalcemia, hyperparathyroidism, risk of osteoporosis, and autoimmune and cardiovascular diseases [3, 5]. Retinoids and vitamin D are routinely used to treat certain diseases and disorders, including age-related macular degeneration, measles, and various skin conditions, such as acne, psoriasis, and atopic dermatitis [6–10]. Previous studies demonstrated that retinoid and vitamin D signaling pathways converge and interact (additively or antagonistically) in response to combined stimulation by both ligands in certain cell types. Additive effects on cellular functions and/or gene expression were observed when inducing differentiation in leukemia [11, 12] and breast cancer cells [13, 14] or inhibiting endothelin-stimulated hypertrophy in rat cardiac myocytes [15]. In contrast, antagonistic effects were observed during the differentiation of keratinocytes [16, 17] and bone resorption [18].

The three RAR isotypes (α, β, and γ) and the VDR are members of the nuclear receptor (NR) superfamily [1]. RARs and VDR activities are predominantly elicited via the formation of dimers with retinoid X receptor (RXR). In contrast to the permissive heterodimers, RXR ligands alone cannot activate RAR-RXR and VDR-RXR dimers [1, 19]. We previously demonstrated that RARα and VDR compete for dimerization with their common partner RXR, in a ligand-dependent manner [20, 21]. Transcriptional responses are induced by the binding of RARs and VDR with their cognate agonists. RARs are activated by all-trans retinoic acid (ATRA), and other retinoic acids (RAs) including 9-*cis-*RA and 9-*cis-*13,14-dihydro-RA, as well as synthetic agonists [22, 23]. VDR is activated by calcitriol (1α,25-dihydroxyvitamin D_3_, 1,25-vitD), some other natural agonists, and synthetic analogs [3, 24]. RARs and VDR activation by their agonists facilitates DNA binding, dimerization with RXR, and the recruitment of a multiprotein coactivator complex [1–3, 25, 26]. The recruited Mediator complex and/or other coactivators increase local histone acetylation, chromatin openness, and the activation of transcriptional machinery at the promoter of the regulated genes. For direct regulation, RAR-RXR and VDR-RXR dimers occupy the regulatory regions localized in proximal promoters and enhancers. Previous studies demonstrated that RARs and VDR can interact at common binding regions of the regulated genes [27–30]. The RAR-RXR and VDR-RXR binding sites are typically composed of two repeats of the hexameric DNA sequence, 5′-AGGTCA-3′, or its variants, which may differ in 1–3 bases from this canonical sequence [2, 3, 31]. Using luciferase reporter assays and electrophoretic mobility shift assays, direct repeats (DRs) with 5 and 3 nucleotide spacers (DR5 and DR3) were originally identified as canonical binding sites for RAR-RXR and VDR-RXR heterodimers, respectively [1, 31]. In addition to DR5, other retinoic acid response elements (RAREs), including DR0, DR1, DR2, and inverted repeat without spacer (IR0), have been identified [32–34]. Global approaches revealed that vitamin D response elements (VDREs) are less variable. DR3 is the dominant VDRE [35–38], and VDR-RXR heterodimers bind to other motifs, including DR2 and DR4, at much lower frequencies [34]. The analysis of individual regulatory regions of 1,25-vitD-responsive genes indicated that additional motifs, such as everted repeats with a six nucleotide spacer (ER6), DR6, and inverted palindromes with a nine nucleotide spacer (IP9), could also be occupied by VDR-RXR [39–41].

The presence of canonical DNA motifs is neither necessary nor sufficient for the binding of NRs or other transcription factors (TFs). Several determinants including chromatin accessibility, binding of other TFs, and DNA methylation also influence the binding of TFs *in vivo*. Global mapping of NR binding sites in a given cell type using chromatin immunoprecipitation coupled with high-throughput sequencing (ChIP-seq) enables the characterization and comparison of different NR binding regions. The pairwise comparisons of cistromes (complete set of binding regions) revealed that the cistromes of various NRs often overlap [42–45]. For example, estrogen receptor (ER) and RARα have 2 365 common binding regions in human MCF7 cells, representing ∼49% of the RARα cistrome [42]. Similarly, 1 989 binding regions, representing ∼56% of the peroxisome proliferator-activated receptor alpha (PPARα) cistrome, overlap with the glucocorticoid receptor (GR) cistrome in murine primary hepatocytes [44].

The aim of this study was to identify and characterize genomic regions occupied by both RARα and VDR in differentiated THP-1 cells. We were especially interested in whether these regions could be involved in the convergence and cooperation of the two pathways. Convergence of pathways refers to the phenomenon in which two or more signaling pathways lead to the same or similar cellular responses by activating or using common downstream modules. In transcriptional events, cooperation is a process in which two or more entities (such as pathways, ligands, or proteins) work together, and the combined action of the entities leads to an increased outcome (such as higher mRNA level, histone modification, RNA polymerase activation, or enhanced cofactor recruitment) relative to the individual effects of each entity. THP-1 is a human leukemia monocytic cell line extensively used to study monocytes and macrophages [46]. THP-1 cells differentiate into macrophage-like cells using phorbol-12-myristate-13-acetate (PMA) and other compounds, including LPS, IL-4, and TGF-β [47–49]. RARs and VDR are expressed in PMA-stimulated THP-1 (PMA-THP-1) cells and stimulation by their cognate ligands induces transcriptional, phenotypic, and functional changes [50, 51]. Moreover, the binding regions and primary target genes of VDR in THP-1 cells and LPS-stimulated THP-1 cells have been defined [35, 36]. Among the three RAR isotypes, we focused on RARα based on the expression level of the three isotypes in THP1 cells and the availability of ChIP-grade antibodies. Instead of ATRA, RARα-specific agonist, AM580, was used to exclude the activation of other RAR isotypes. Notably, our investigation does not rule out the potential involvement of other RAR isotypes in the interaction of retinoids and vitamin D signaling in this cell line. We performed ChIP-seq, RNA-sequencing (RNA-seq), and assay for transposase-accessible chromatin with high-throughput sequencing (ATAC-seq) experiments and used bioinformatics tools to map and characterize the common binding regions of RARα and VDR. We identified a large subset of regions (dominantly localized to highly accessible chromatin regions) occupied by both RARα and VDR in ligand-stimulated PMA-THP-1 cells. Correlation analyses of ChIP-seq data and transcriptional programs indicated that the common binding regions were involved in regulating genes induced by both RARα and VDR agonists. Our findings suggest that the common binding regions of RARα and VDR are involved in the convergence and cooperation of retinoid and vitamin D signaling pathways.

## Materials and methods

### Cell culture and stimulations

The human monocytic cell line, THP-1, was obtained from the American Type Culture Collection. Cells were cultured in a humidified atmosphere at 37°C and 5% CO_2_ in RPMI-1640 medium (Gibco) supplemented with 10% fetal bovine serum (Biosera), 0.05 mM 2-mercaptoethanol (Gibco), 1% penicillin-streptomycin solution (Sigma-Aldrich), and 1 mM sodium-pyruvate (Gibco). Cells were passaged every 3 days, and the cell density after each passage was approximately 250 000 cells/ml. Cells were stimulated with 20 nM PMA (Sigma-Aldrich) for 16 h to differentiate THP-1 cells into macrophage-like cells (PMA-THP-1 cells). After differentiation, PMA-THP-1 cells were maintained in phenol red-free RPMI medium (Gibco) supplemented with 10% charcoal-stripped fetal bovine serum of South American origin (Biowest) and 1% penicillin-streptomycin solution for 30 min. Cells were then stimulated with 1:1 Dimethyl Sulfoxide (DMSO)–ethanol (vehicle control), the specific RARα agonist (100 nM AM580, BioVision), 1,25-vitD (100 nM, Sigma-Aldrich), or a combination of the two agonists. Cells were harvested at different times depending on the experiment.

### RNA-seq experiments and data analysis

For RNA-seq experiments, PMA-THP-1 cells were stimulated for 6 h with vehicle or ligands, and total RNA was isolated using a Quick-RNA Miniprep Kit (ZYMO research). The quality of RNA was verified on an Agilent BioAnalyzer using a Eukaryotic Total RNA Nano Kit. RNA-seq libraries were prepared from total RNA using an Ultra II RNA Sample Prep kit (New England BioLabs). Sequencing was performed on the Illumina NextSeq 500 platform using single-end 75-cycle sequencing. Twelve RNA-seq datasets were generated, including three replicates each of vehicle, AM580, 1,25-vitD, and combined agonist stimulations. Sequence reads were aligned to the hg38 genome assembly using HISAT2. For subsequent analyses, Binary Alignment Map (BAM) files were imported to the Strand NGS program (Strand Life Sciences Pvt., Bangalore, India). The workflow to identify differentially expressed genes was performed in three steps. First, genes expressed at low levels in all conditions were excluded. Then, ANOVA with the Benjamini–Hochberg procedure (adjusted *P* < 0.05) was performed. The differentially expressed genes were determined by Tukey posthoc tests (*P* < 0.05), and the results were filtered using 1.5 and 0.66 cut-offs for up- and downregulated genes. A Gene Transfer Format (GTF) file (GRCh38.p14) was used to determine the transcription start site (TSS) of the genes.

Using the following four steps, cooperatively upregulated protein-coding genes were determined. First, we identified the more effective single ligand for all genes upregulated by AM580 or 1,25-vitD. Second, using triplicates from the RNA-seq dataset, we identified genes that showed significant differences between the more effective single ligand and the combined treatment (t-test, *P* ≤ 0.05). Third, we identified genes upregulated in a cooperative manner, by calculating the ratios of combined *versus* the more effective single ligands and using a cut-off of 1.5. Finally, the protein-coding genes were determined using the annotation information on gene biotypes in the GTF file. Heatmaps were generated using DisplayR web-based tool and the “pheatmap” package in R (version 1.0.12). For some heatmaps, the values were row-normalized, allowing for independent adjustment of the values within each row.

### Real-time quantitative polymerase chain reaction (RT-qPCR)

The messenger RNA (mRNA) expression of selected target genes, including *PTGES*, *CAMP*, *FBP1, HBEGF, RAB20*, and *TGM2*, and the enhancer RNA (eRNA) transcripts of selected binding regions were measured using RT-qPCR. PMA-THP-1 cells were stimulated with vehicle or ligands for 1–6 h to measure mRNA levels and 6 h to measure eRNA. Total RNA was isolated using a Quick-RNA Miniprep Kit (ZYMO research). RT-qPCR was performed using the RevertAid First Strand complementary DNA (cDNA) Synthesis kit (Thermo Scientific) and the LightCycler 480 SYBR Green I Master (Roche). Relative RNA expression was quantified using the comparative threshold cycle method and normalized to cyclophilin A (*PPIA*) expression. Biological triplicates were included in all experiments. The list of RT-qPCR primers for mRNA and eRNA measurements is provided in Supplementary Table S1.

### ChIP-seq experiments

ChIP-seq was performed, as previously described [52–54]. Briefly, 7–10 million differentiated THP-1 cells were stimulated with the vehicle or ligand for 1–2 h. The cells were cross-linked with 2 mM disuccinimidyl glutarate (Sigma-Aldrich) for 40 min and 1% methanol-free formaldehyde (Thermo Fisher Scientific) for 10 min. Cross-linking was terminated by adding 0.125 M glycine (Sigma-Aldrich) for 10 min. The ChIP lysis buffer (150 mM NaCl, 1 mM EDTA pH 8, 20 mM Tris–HCl pH 8, 1% Triton X-100, and 0.1% SDS was supplemented with protease inhibitors (cOmplete Mini EDTA-free protease inhibitor cocktail, Roche). The chromatin was sheared by sonication (Diagenode Bioruptor Standard) and immunoprecipitated overnight using antibodies against RARα (ab41934, Abcam), VDR (ab109234, Abcam) and Mediator complex subunit 1 (MED1) (A300-793A, Bethyl). Chromatin-antibody complexes were pulled down with magnetic beads (Protein A or G Dynabeads, Thermo Fisher Scientific), washed, and eluted. Eluted complexes were de-crosslinked overnight and purified using NucleoSpin Gel and a PCR Clean-up Kit (Macherey-Nagel). ChIP-DNA was quantified using a Qubit fluorimeter. Indexed cDNA libraries were prepared from 1 to 10 ng of ChIP-DNA using a TruSeq ChIP Sample Preparation Kit (Illumina) according to the manufacturer's instructions. Libraries were sequenced on the Illumina NextSeq 500 platform using single-end 75-cycle sequencing. The following 20 ChIP-seq datasets were generated: two replicates of VDR ChIP-seq from PMA-THP-1 cells stimulated with vehicle or 1,25-vitD (*n* = 4), two replicates of RARα ChIP-seq from PMA-THP-1 cells stimulated with vehicle or AM580 (*n* = 4), and three replicates of MED1 ChIP-seq from PMA-THP-1 cells stimulated with vehicle, AM580, 1,25-vitD or combined ligands (*n* = 12).

### Primary ChIP-seq data analysis, peak calling and normalization

The ChIP-seq data were analyzed using our ChIP-seq analysis pipeline [55], as described previously [54]. Model-based analysis of ChIP-seq version 2 (MACS2) [56] was used for predicting summits of the “peaks” using the following specific parameters: q value cutoff (*q*) = 0.001 and subpeaks deconvolved within each peak (–call-summits). Artifacts were removed using the ENCODE blacklist [57]. Regions with accession prefixes of NW and NT were excluded; only regions starting with NC (complete genomic assembly) were used for the analyses. The identified peak summits were extended by ± 100 bp to obtain binding regions. If two summits in the same dataset were closer than 200 bp, the two peaks were merged and the center of the two summits was considered the new summit. Normalized tag counts (expressed as reads per kilobase per million mapped reads, RPKM) were calculated using bamtools, bedtools, and awk. Normalized coverage values were calculated by dividing the occupancy values (RPKM) by the 90^th^ percentile of all RPKM values for the corresponding dataset. Integrative Genomics Viewer (IGV; Broad Institute [58]) was used for data browsing and creating representative snapshots. The values in the genome coverage files (BedGraphs) were normalized and converted into Tile Data Format (TDF) files using igvtools with the “toTDF” option. Read distribution (RD) plots were generated by annotatePeaks.pl (HOMER) [59] using tag directories and bed files. The histograms were visualized with Java TreeView. Peaks were associated with the TSS of the nearest gene, and the peak-to-TSS distance was calculated using the annotatePeaks.pl (Homer), incorporating the gtf-version 2.2 (genome-build GRCh38.p14, NCBI Assembly:GCF_000001405.40).

### ATAC-seq experiments, primary data analysis, peak calling, normalization, and statistical analysis

ATAC-seq was performed, as previously described with minor modifications [60]. PMA-THP-1 cells were stimulated with vehicle or AM580 for 1 h in biological duplicates. After collecting 50 000 cells in ice-cold PBS, the nuclei were isolated with ATAC lysis buffer (10 mM Tris–HCl pH 7.4, 10 mM NaCl, 3 mM MgCl_2_, and 0.1% IGEPAL). Tagmentation of the nuclei was performed using the Tagment DNA TDE1 Enzyme and Buffer Kits (Illumina). Indexing was performed using the Nextera DNA library preparation kit (Illumina). After tagmentation and indexing, DNA was purified with a NucleoSpin Gel and PCR Clean-up Kit (Macherey-Nagel). Indexed DNA was amplified with Kapa Hifi Hot Start Kit (Kapa Biosystems) in nine PCR cycles. Amplified libraries were purified again with a NucleoSpin Gel and PCR Clean-up Kit (Macherey-Nagel). Fragment distribution of libraries was assessed with an Agilent Bioanalyzer and libraries were sequenced on an Illumina NextSeq 500 platform. Similar to ChIP-seq analyses, the ATAC-seq raw reads were analyzed using an analysis command line pipeline. Briefly, Burrows-Wheeler Alignment (BWA) [61] was used to align the reads with the hg38 genome assembly using default parameters and MACS2 was used for predicting ATAC-seq peaks (q-value ≤0.001). Artifacts were removed using the ENCODE blacklist. Regions with accession prefixes of NW and NT were excluded; only regions starting with NC (complete genomic assembly) were used for the analyses. For visualization, genome coverage files (BedGraphs) were generated with makeUCSCfile.pl and converted into TDF files using igvtools with the “toTDF” option. IGV was used for data browsing and creating representative snapshots. RD plots with ATAC-seq signals were generated, as previously described (see ChIP-seq data analysis). As a control set, we used randomly selected, size-matched genomic regions starting with NC (complete genomic assembly) (*n* = 5 000). An R Bioconductor package, DiffBind (version 3.19) with edgeR tool was used to identify the peaks with significantly different signals false discovery rate (FDR ≤ 0.05) by ligand treatment [62]. Volcano plots showing the comparisons of ATAC-seq signals in vehicle *versus* AM580-treated samples (fold change (FC) and FDR) were generated by DiffBind.

### Determination of consensus peak sets

The identified peaks (summits ± 100 bp) were used to determine the consensus peak sets. A peak was classified as a consensus peak if it was identified in both replicates. For this analysis, MACS2 “narrow peaks” were also included, as some regions did not have summits determined by the algorithm in one replicate, despite being detected as “narrow peaks” in the same replicate. Therefore, peaks with a summit in one replicate and a “narrow peak” in the other were also considered as consensus peaks. In the case of MED1 ChIP-seq datasets with three replicates, consensus peak sets were determined using DiffBind. For each MED1 ChIP-seq sample, summits were defined using the MACS2 tool and extended by ± 100 bp. These regions, along with the BAM files, were used as input for DiffBind with the default settings (minOverlap = 2). Using this approach, a peak was included in the consensus set if it was present in at least two replicates. The use of three replicates of MED1 allowed for more accurate statistical analysis in identifying ligand-induced signals.

### Analysis of overlap between genomic regions and ligand-induced changes in ChIP-seq datasets

To determine the overlap between ChIP-seq peaks in the cells treated with vehicle and ligand(s), and also between the datasets from different ChIP-seq factors (e.g. RARα versus MED1) and different omics data (e.g. RARα ChIP-seq versus ATAC-seq), intersectBed (bedtools) was used in two steps. First, we identified a subset in one dataset that overlapped with regions in the other dataset. Second, we identified the non-overlapping subsets for each of the two datasets. Notably, the total number of peaks in the subsets matches the original set only for the first input file when the overlapping subset is determined. For the comparison of consensus RARα (AM580) and VDR (1,25-vitD) peak sets, we used a different approach. This method treated the RARα and VDR consensus sets equally, ensuring no preference was given to either set. We merged the RARα and VDR regions from both consensus peak sets using mergeBed (bedtools), labeling the merged regions as common peaks.

An R Bioconductor package, DiffBind (version 3.19) with edgeR tool was used to identify the peaks with significantly different signals (FDR ≤ 0.05) by ligand treatment [62]. Volcano plots showing the comparisons of RARα, VDR or MED1 signals in vehicle *versus* ligand-treated samples (FC and FDR) were generated by DiffBind. Heatmaps showing various kinds of ChIP-seq data analysis were generated as described in the “RNA-seq experiments and data analysis” section.

Using DiffBind, we identified a merged peak set from 12 MED1 ChIP-seq samples (minOverlap = 3). The MED1 occupancy value (referred to as the MED1 signal) was calculated for all consensus MED1 peaks in all samples. We then compared MED1 signals (combined vs. single treatments) by calculating FCs, identifying the more effective single treatment, and performing statistical analyses (unpaired t-test) to determine p-values. This approach allowed us to identify MED1 peaks that exhibited a significantly increased signal in the combined treatment compared to the more effective single treatment (FC > 1, *P* ≤ 0.05). Finally, we identified RARα and VDR binding regions belonging to various binding clusters that were colocalized with the peaks showing significantly increased MED1 signals (both globally and within the TSS ± 25 kb of cooperatively upregulated genes).

### Terminology for genomic regions

Genomic regions identified by the MACS2 peak-calling algorithm are referred to as “peaks” or “binding regions”. The size of binding regions for RARα-only and VDR-only clusters were uniformly 200 bp. The regions in the common set were larger because they were generated by merging two or more RARα and VDR binding regions. The “peak” term also refers to a geometrical shape representing the occupancy of proteins in genome browser tracks. All binding regions for a given TF were collectively called the “cistrome”. The term “binding site” was restricted to short genomic regions, where direct physical interaction between TFs and DNA occurs. TSS positions of RefSeq genes were obtained from the University of California, Santa Cruz (UCSC) database.

### DNA motif analysis

The term “DNA motif” refers to a set of aligned sequences summarized by position weight matrices (PWMs) and visualized using motif logos. To identify the most enriched DNA motifs in the top 1 000 RARα (AM580) and VDR (1,25-vitD) ChIP-seq peaks, *de novo* motif discovery was performed using HOMER tool (findMotifsGenome.pl) with the following parameters: motif lengths of 17 and 18 bp for RARα peaks, 15 and 16 bp for VDR peaks, and a region size of 200 bp.

The occurrence of PWMs was evaluated in the various peak sets in six steps. (i) PWMs were obtained from HOMER and Jaspar databases (http://homer.ucsd.edu/homer/motif/HomerMotifDB/homerResults.html and https://jaspar2018.genereg.net/collection/core/). The selected PWMs are as follows: VDR-DR3 (GSE22484/Homer, motif 394), RARα-DR5 (MA0730.1/Jaspar), ERE-IR3 (MA0112.3/Jaspar), GRE-IR3 (GSE32465/Homer, motif 145), LXR-DR4 (GSE21512/Homer, motif 204), and PPARα-DR1 (GSE47954/Homer, motif 290). (ii) The PWM alignment scores were calculated using control sets containing randomly selected, size-matched genomic sequences from regions starting with NC (complete genomic assembly) (*n* = 5 000). Alignment scores, which reflect the similarity of sequences to the PWM, were determined using annotatePeaks.pl (HOMER). The score thresholds (cut-offs) were defined as the scores giving 5% positive matches in the control set. (iii) The binding regions in the RARα, VDR, and other cistromes with alignment scores above the thresholds were filtered and used for *de novo* motif discovery (findMotifsGenome.pl, HOMER). Regions with accession prefixes of NW and NT were excluded; only regions starting with NC (complete genomic assembly) were used for the analyses. (iv) Thresholds were calculated for the PWMs obtained from the *de novo* motif discovery using a control set (see step ii). (v) DNA motifs in the entire human genome were mapped using the PWMs and the computed score thresholds in scanMotifGenomeWide.pl (HOMER). The genomic positions of DR1, DR2, and DR5 motifs were merged to generate a file containing the positions of all putative RAREs. (vi) The prevalences of motifs in cistromes and binding clusters were determined using intersectBed (bedtools), and files with the positions of binding regions and motifs were used for the calculations.

PWMs for DR0, DR1, DR2, DR4, and DR6-DR9 were generated by modifying the spacer bases of the PWM of DR5. PWMs for IR0-IR9 were generated by modifying the spacer bases of the PWM of DR5 and by changing the orientation of one half-site. Distances between motifs were determined using closestBed (bedtools, with -d switch). Histograms from distance values were generated using ggplot2. Logos from PWMs were created by motif2Logo.pl (HOMER). Circos plots (phyton) [63] were used to display the overlap of cistromes and the fraction of regions containing two types of motifs.

## Results

### RARα and VDR binding regions overlap in PMA-THP-1 cells

**Figure 1.**
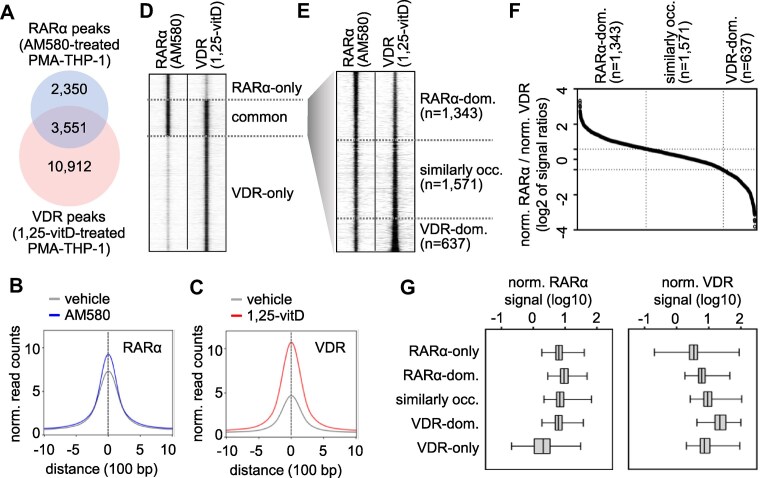
Mapping common binding regions of RARα and VDR in PMA-stimulated THP-1 cells (PMA-THP-1). (**A**) The overlap of genomic regions occupied by RARα and VDR in PMA-THP-1, as determined by ChIP-seq. The RARα and VDR binding regions were determined in PMA-THP-1 treated for 1 h with 100 nM AM580 and 100 nM 1α,25-dihydroxyvitamin D_3_ (1,25-vitD), respectively. (B and C) The normalized read counts in peak summit ± 1 000 bp of RARα (**B**) and VDR (**C**) cistromes in vehicle- and ligand-stimulated cells. (**D**) A RD plot showing the RARα and VDR ChIP-seq signals in 2 kb windows. (**E**) A RD plot indicating the RARα and VDR ChIP-seq signals in three clusters of common binding regions in 2 kb windows. RARα-dom., RARα-dominant; occ., occupied; and VDR-dom., VDR-dominant. Clusters were determined as shown on panel F. (**F**) The ratio of normalized RARα and VDR signals in the regions occupied by both RARα and VDR. Binding regions were ranked based on the ratios of RARα to VDR normalized occupancies. Using 1.5 and 0.66 cut-off values, three clusters of binding regions were determined: RARα-dominant (left), similarly occupied (middle), and VDR-dominant (right). (**G**) Box-and-whisker plots demonstrating the normalized RARα and VDR signals in the five binding clusters..

The binding regions of RARα and VDR were determined using ChIP-seq in PMA-THP-1 cells stimulated for 1 hour with vehicle or selective agonists, AM580 and 1,25-vitD (Fig. 1A–C and Supplementary Fig. S1). The two replicates of ChIP-seq peaks for RARα in AM580-stimulated cells and VDR in 1,25-vitD-stimulated cells were compared. The consensus peak sets of RARα and VDR contained 5 936 and 14 505 peaks, respectively (Supplementary Fig. S1A and D). The RARα and VDR peak sets in ligand-stimulated cells were also compared with those in vehicle-stimulated cells (Supplementary Fig. S1B–C and S1E–F) and with other previously published peak sets (Supplementary Fig. S2) [36, 64, 65]. We found that agonists increased both the number of binding regions and the occupancy values, especially for VDR (Fig. 1B and C and Supplementary Fig. S1B and E). Differentially occupied regions upon ligand stimulation comprised a higher proportion of consensus peaks in the case of VDR than for RARα (Supplementary Fig. S1C and F).

We observed that the two cistromes largely overlapped in PMA-THP-1 cells treated with their cognate ligands (Fig. 1A). In this way, the genomic regions that were occupied either by RARα or VDR in PMA-THP-1 cells could be grouped into three categories: RARα-only (∼14%), common (∼21%), and VDR-only (∼65%). The 3551 common regions for RARα and VDR represented ∼60% and ∼24% of the RARα and VDR cistromes, respectively (Fig. 1A and D). The common binding regions were grouped based on the occupancy signal intensities. The RARα and VDR signals were normalized, and the ratio between the normalized RARα and VDR occupancy values was calculated (Fig. 1E–F). Using 1.5 and 0.66 cut-off values, the regions occupied by both RARα and VDR were classified into RARα-dominant (*n* = 1 343), similarly occupied (*n* = 1 571), and VDR-dominant (*n* = 637) regions (Fig. 1E–F). Five clusters, including the three clusters of common binding regions and the two specific clusters (RARα-only and VDR-only), were used for subsequent analyses (Supplementary Table S2). The RARα and VDR occupancy values in the five clusters were determined. The occupancy values in the common binding regions were similar to or higher than the RARα-only and VDR-only clusters (Fig. 1G).

### RARα and VDR up-regulate overlapping sets of genes in PMA-THP-1

**Figure 2.**
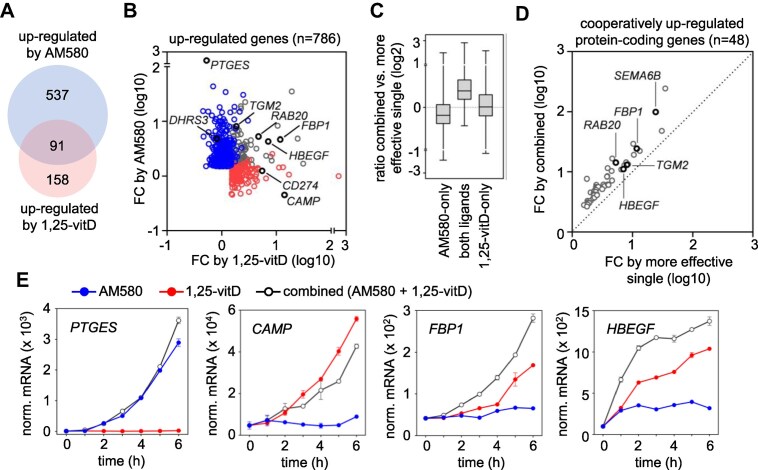
Identification of genes upregulated by RARα and/or VDR agonists in PMA-THP-1 cells. (**A**) The overlap of gene sets upregulated by RARα and VDR agonists in PMA-THP-1, as determined by RNA-seq. Cells were stimulated by agonists or vehicle for 6 hours. (**B**) The FCs induced by AM580 and 1,25-vitD are shown in a scatter plot. Gene sets are color-coded as follows: upregulated by AM580-only (blue), by both ligands (grey); and by 1,25-vitD-only (red). Representative genes for each gene set are indicated on the plot. (**C**) Box-and-whisker plot showing the effects of the combined treatments over the more effective single treatments. The ratios of FCs induced by combined treatment with AM580 and 1,25-vitD versus the more effective single treatments were calculated for each gene. (**D**) A scatter plot showing fold induction values of cooperatively upregulated protein-coding genes by the combined treatment versus the more effective single ligand. For each gene, the more effective single ligand was selected by comparing mRNA levels from samples treated with either AM580 or 1,25-vitD. The mRNA levels corresponding to the more effective ligand were then compared to the levels observed in cells treated with a combination of both ligands. Representative cooperatively upregulated genes are indicated on the plot. (**E**) Time courses of the mRNA expression of four representative genes measured by RT-qPCR in PMA-THP-1 treated with AM580, 1,25-vitD, or both agonists (combined). Expression levels were normalized to *PPIA*. One representative experiment out of three is shown. Values are expressed as the mean of technical triplicates ± SD of the mean.

We evaluated the relationship between the binding of these receptors and gene regulation. For the correlation analysis, we first identified the genes regulated by AM580 and 1,25-vitD using RNA-seq. The number of up- or downregulated genes were as follows: AM580, *n* = 1 094; 1,25-vitD, *n* = 387; and combined treatment, *n* = 1 507. Differentially expressed gene lists are provided in Supplementary Table S3 and were compared in Supplementary Fig. S3A and B. We also compared the lists of upregulated genes by AM580 and 1,25-vitD and determined the following sets: upregulated by AM580-only (*n* = 537), upregulated by both ligands (*n* = 91), and upregulated by 1,25-vitD-only (*n* = 158) (Fig. 2A and B and Supplementary Fig. S3C). The upregulated genes included previously identified retinoid- and vitamin D-responsive genes, for some of which the response elements were characterized [66–77]. These genes included *PTGES* and *DHRS3* (upregulated by AM580-only), *CAMP* and *CD274* (upregulated by 1,25-vitD-only), and *FBP1*, *HBEGF*, *TGM2*, and *RAB20* (upregulated by both ligands) (Fig. 2B). The ratios of the FCs induced by the combined treatment versus the more effective single ligand were also calculated. The results indicated that many commonly regulated genes were induced in a cooperative manner upon combined treatment (Fig. 2C and Supplementary Fig. S3D). We identified 57 genes including 48 protein-coding genes that were cooperatively upregulated by the two ligands (Fig. 2D and Supplementary Table S3). Notably, many of these genes (*n* = 22) were upregulated exclusively by either AM580 or 1,25-vitD but they exhibited cooperative regulation. The mRNA expression time courses for representative genes were determined using RT-qPCR. These results indicated that the cooperation between the two ligands was detected at early time points (2–3 h) in the expression of commonly regulated representative genes (*FBP1*, *HBEGF*, *RAB20*, and *TGM2*) (Fig. 2E and Supplementary Fig. S3E).

### Common binding regions are frequently (but not exclusively) annotated with genes upregulated by both AM580 and 1,25-vitD

**Figure 3.**
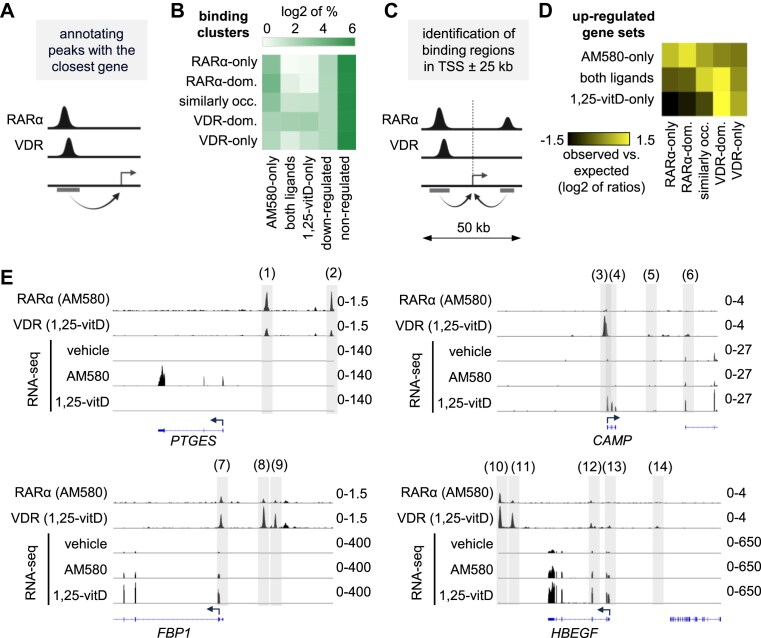
The relationship between gene induction and occupancy of RARα and VDR. (**A**) A schematic view of peak annotation. (**B**) A heatmap showing the proportion of various gene sets identified as the closest genes to RARα and VDR binding clusters. The closest gene was determined for all peaks and the proportions of various gene sets were calculated separately for each binding cluster. (**C**) A schematic view of the approach to map binding regions in the TSS ± 25 kb of the upregulated genes. (**D**) The enrichment of binding region clusters in the TSS ± 25 kb of genes belonging to the three gene sets: upregulated by AM580-only, upregulated by both ligands, and upregulated by 1,25-vitD-only. Enrichment was calculated by dividing the actual number of occurrences by the expected number of occurrences (observed/expected) for each category. (**E**) IGV snapshots showing representative genes upregulated by AM580-only (*PTGES*), 1,25-vitD-only (*CAMP*), or by both ligands (*FBP1* and *HBEGF*) in PMA-THP-1. The tracks display normalized ChIP-seq and RNA-seq data. The binding regions in TSS ± 25 kb belong to the following clusters: RARα-dominant [1, 2], similarly occupied [12], VDR-dominant [4, 7, 8, 10, 11], and VDR-only [3, 5, 6, 9, 13, 14].

The relationship between gene induction and the binding patterns of RARα and VDR was evaluated using two approaches. First, the binding regions were annotated with the closest gene and the regulation of the annotated genes was investigated (Fig. 3A and Supplementary Table S2). In ∼56% of the RARα- and/or VDR-occupied regions, the distance to the closest gene was less than 20 kb, consistent with a previous study [33] (Supplementary Fig. S4A). Most of the binding regions in the five clusters (79–89%) were annotated with genes that were not regulated (Fig. 3B). Notably, the rest of the binding regions were annotated with both upregulated and downregulated genes. In the binding regions that were annotated with upregulated genes, the binding pattern of RARα and VDR correlated with gene induction by the cognate ligands. The RARα-only and RARα-dominant binding clusters were annotated more frequently with genes upregulated only by AM580 compared with the other binding clusters. Similarly occupied binding regions were annotated with all three types of upregulated genes. The VDR-only and VDR-dominant binding regions were annotated more frequently with genes upregulated only by 1,25-vitD compared with other binding clusters (Fig. 3B). We found that a considerable fraction of the VDR-only (3.7%) and VDR-dominant (4.3%) clusters were associated with genes regulated only by AM580 (Fig. 3B). The fold induction values by AM580 were the highest for the genes annotated to RARα-only and RARα-dominant regions. Similarly, the fold induction values by 1,25-vitD were the highest for genes annotated to VDR-only and VDR-dominant regions (Supplementary Fig. S4B).

In the second approach, the enrichment of different types of binding regions was assessed in the TSS ± 25 kb window of upregulated genes (Fig. 3C and Supplementary Table S4). The number of binding regions in TSS ± 25 kb for genes regulated by both ligands was higher than the number of binding regions for the other two gene sets (Supplementary Fig. S4C). The observed versus expected ratios were calculated to determine the representation of binding clusters in the TSS ± 25 kb window of various upregulated gene sets (Fig. 3D). The ratios were calculated by dividing the actual number of occurrences by the number of expected occurrences. (The expected occurrences were calculated from the number of all identified binding regions and the size of the clusters). The enrichment analysis and representative upregulated genes are shown in Fig. 3D–E. The RARα-only and RARα-dominant binding regions were over-represented in the TSS ± 25 kb of genes upregulated by AM580-only (Fig. 3D). The VDR-only and VDR-dominant binding regions were over-represented in the TSS ± 25 kb of genes upregulated by 1,25-vitD-only (Fig. 3D). The similarly occupied and VDR-dominant binding regions were over-represented in TSS ± 25 kb regions of genes upregulated by both agonists (Fig. 3D). Collectively, our results indicated that common binding regions play an important role in the regulation of genes induced by both ligands. However, they were not exclusively annotated with such upregulated genes, suggesting that common binding did not necessarily correlate with the regulatory capacity of both receptors.

### MED1 is recruited in a ligand-responsive manner at many RARα and VDR common binding regions.

The activity of gene regulatory regions can be assessed by various measurements, including histone tail acetylation, co-activator occupancy, and enhancer RNA (eRNA) expression. In this study, the genome-wide occupancy of Mediator complex subunit 1 (MED1) was determined using ChIP-seq and eRNA expression was analyzed in selected binding regions using RT-qPCR.

MED1 (also known as TRAP220 or DRAP205) is a subunit of the Mediator complex, which is recruited by many NRs, including RARs and VDR [78–81]. The Mediator complex participates in the promotion of the preinitiation complex assembly and phosphorylation of RNA Polymerase II [82]. High MED1 levels are associated with super-enhancers and regions occupied by multiple TFs [83–85]. We concluded that MED1 could be used to evaluate the activity of binding regions. ChIP-seq experiments were conducted using an antibody against MED1 in PMA-THP-1 treated with vehicle, AM580, 1,25-vitD, or combined agonists for 2 h. We could identify 36 629 and 33 645 MED1-occupied regions in cells treated with AM580 and 1,25-vitD, respectively (Supplementary Fig. S5A-B). The regions where significant differences between the conditions with respect to the MED1 signals were identified (Supplementary Fig. S5), were used for five types of analyses.

**Figure 4.**
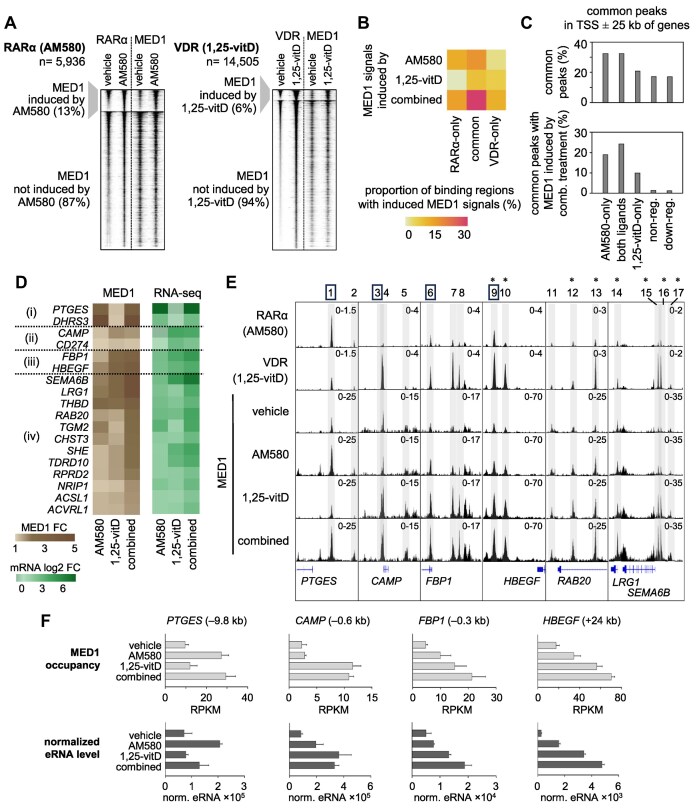
Evaluation of the activity of RARα and VDR binding regions by measuring MED1 occupancy genome-wide and eRNA levels at representative binding regions (**A**) RD plots show the colocalization of MED1 with RARα (left) and VDR (right) peaks in vehicle- or ligand-stimulated cells within 2 kb window. The binding regions of RARα and VDR were classified based on the MED1 induction after ligand treatment. Significantly increased MED1 signals (DiffBind FDR ≤ 0.05) were detected in the case of 13% and 6% of all RARα (AM580) and VDR (1,25-vitD) peaks, respectively. (**B**) The proportion of RARα-only and VDR-only clusters, as well as common binding regions (RARα-dominant, similarly occupied, VDR-dominant) with significant induced MED1 signal (DiffBind, FDR ≤ 0.05) upon treatment with AM580, 1,25-vitD or both ligands (combined). (**C**) Prevalence of common binding regions in the TSS ± 25 kb of genes belonging to various gene sets. The graphs show the prevalence of all common peaks (top), and common peaks with MED1 signals significantly increased (DiffBind, FDR ≤ 0.05) by combined (comb.) treatment (AM580 + 1,25-vitD) (bottom). The peak counts were normalized by the total number of peaks in the regions. Non-reg., non-regulated; down-reg., downregulated. (**D**) Heatmaps displaying fold induction values of MED1 occupancy (left) and log 2 fold induction values of mRNA levels (right) for representative genes. The representative genes include genes upregulated by (i) AM580 only, (ii) 1,25-vitD only, or (iii) both ligands. The data for several genes that were cooperatively upregulated are also displayed (iv). The MED1 occupancy was calculated within TSS ± 25 kb of genes in ChIP-seq datasets from cells treated with vehicle, AM580, 1,25-vitD, or combined ligands (AM580 + 1,25-vitD). (**E**) IGV snapshots showing representative genes upregulated by AM580-only (*PTGES*), 1,25-vitD-only (*CAMP*), or by both ligands in a cooperative manner (*FBP1 and HBEGF, RAB20, LRG1 and SEMA6B*). The tracks display RARα, VDR, and MED1 ChIP-seq data. The highlighted binding regions belong to RARα-dominant [1, 2], similarly occupied [11, 14], VDR-dominant [4, 6, 7, 9, 10, 12, 13, 15, 16], and VDR-only [3, 5, 8, 17]. The regions selected for eRNA measurements are indicated by squares. Binding regions that were co-localized with MED1 peaks, where the signals were significantly higher (*P* ≤ 0.05) in samples treated with both ligands (combined, comb.) than in samples treated with any single ligand, are indicated by asterisks. For statistical analysis, the more effective single ligand for each binding region was determined, and the signal values corresponding to that ligand were used for the calculation. (**F**) The MED1 occupancy and normalized (norm.) eRNA expression at representative binding sites as measured by ChIP-seq and RT-qPCR, respectively. The MED1 occupancy for each region is expressed as the mean RPKM ± SD of three replicates of samples treated with vehicle or agonist(s) for 2 h. The eRNA expression was measured after a 6-h treatment with vehicle or agonist(s) and were normalized to *PPIA* mRNA expression. One representative experiment out of three is shown and values are expressed as the means of technical triplicates ± SD.

First, the overlap between various RARα/VDR and MED1 peak sets was determined. We found that most RARα and VDR peaks (84% and 63%, respectively) were overlapping with MED1 peaks (Supplementary Fig. S5A and B). Notably, MED1 was recruited in a ligand-responsive manner at only 13% of RARα peaks (AM580-treated cells) and 6% of VDR peaks (1,25-vitD-treated cells) (Fig. 4A). Second, we determined the regions belonging to various binding clusters where MED1 was recruited in a ligand-dependent manner (Fig. 4B and Supplementary Fig. S5F). The regions where MED1 was recruited in a ligand-responsive manner (MED1 signals induced by AM580, 1,25-vitD, or combined treatment) were more frequent in the common binding cluster than in the RARα-only and VDR-only clusters. Notably, 26% of peaks in the common clusters were associated with induced MED1 signals in response to combined treatment compared with 12% for RARα-only and 10% for VDR-only clusters (Fig. 4B). Third, the common binding regions associated with various gene sets were analysed with respect to the MED1 signals (Fig. 4C). The analyzed gene sets included three upregulated gene sets (AM580-only, both ligands, and 1,25-vitD-only, Fig. 2A), non-regulated highly expressed genes (*n* = 250), and downregulated genes (*n* = 559). The counts of common peaks were normalized by the total counts of all peaks detected within TSS ± 25 kb of the genes in the analyzed sets. When analyzing the enrichment of common binding regions regardless of MED1 signals, a similar or only slightly higher enrichment was detected in genes upregulated by both ligands compared to the other gene sets (Fig. 4C, upper chart). Notably, considerable differences emerged when analysing common binding regions where MED1 signals were significantly induced by the combined treatment relative to the vehicle (Fig. 4C, lower chart). These common regions were almost absent in non-regulated and downregulated genes and their prevalence was higher in the gene set upregulated by both ligands compared to the AM580-only and 1,25-vitD-only gene sets. Fourth, we investigated MED1 binding within TSS ± 25 kb of the protein-coding genes that were upregulated cooperatively by the two ligands. (The fold induction of these 48 genes by ligand treatments is shown in Fig. 2D.) We found that overall MED1 signals were higher in samples treated with the combined ligands compared to those treated with AM580 or 1,25-vitD, within ± 25 kb of many protein-coding genes that were cooperatively upregulated (Fig. 4D and Supplementary Fig. S5G).

Fifth, we analysed the RARα and VDR binding regions where MED1 was recruited cooperatively upon combined treatment (AM580 + 1,25-vitD). We found that the regions where MED1 was recruited cooperatively overlapped with regions belonging to all three binding categories (RARα-only, common and VDR-only). We determined the proportions of binding categories in the cooperative MED1 subset overlapping with RARα or VDR peaks (Supplementary Fig. S5H). We found the prevalences of binding categories in this MED1 subset and the merged cistrome (Fig. 1A) differed. The common binding category was overrepresented (43% versus 21%), while the other two categories were underrepresented (8% versus 14% for RARα-only, and 49% versus 65% for VDR-only) among the regions where MED1 was recruited cooperatively upon combined treatment. We were interested in how single and combined ligand treatments induce the MED1 recruitment at binding regions localized within TSS ± 25 kb of cooperatively upregulated genes. In total, 104 RARα and VDR binding regions were localized within TSS ± 25 kb of cooperatively upregulated genes (Supplementary Fig. S5I). Among them, in 11 regions (mainly common binding regions), MED1 was recruited cooperatively upon combined treatment (Supplementary Table S4). The cooperation was determined by comparing the MED1 signals (combined versus single treatments) by performing statistical analyses (t-test, *P* ≤ 0.05). Notably, in many cases, we observed that the MED1 signal was higher with combined treatment compared to the more effective single treatment, but the difference was not statistically significant (Supplementary Fig. S5I). ChIP-seq data for representative genomic regions annotated with cooperatively upregulated genes, as well as AM580-specific and 1,25-vitD-specific genes, are shown in Fig. 4E.

These results together suggest that common binding regions are involved in the cooperation between retinoid and vitamin D pathways. However, this conclusion does not imply that all common regions are involved in the cooperation, nor does it rule out the possibility that non-common regions and even non-genomic mechanisms may also play a role in cooperation.

After the global analysis of MED1 datasets, eRNA expression in a selected set of binding regions was measured using RT-qPCR after 6 h of stimulation (Fig. 4F). The transcription of non-coding eRNAs is a feature of active enhancers, and the magnitude of eRNAs usually reflects enhancer activity [86]. We studied four binding regions annotated with genes (*PTGES*, *CAMP*, *FBP1*, and *HBEGF*) that were investigated in our previous analyses (Fig. 4E). In two common regions, eRNA expression was induced by AM580 or 1,25-vitD stimulation, and cooperation between the two ligands was observed. These common binding regions (both VDR-dominant) were annotated with *FBP1* (TSS - 0.3 kb) and *HBEGF* (TSS + 24 kb). The third common region (RARα-dominant) was annotated with *PTGES* (TSS - 9.8 kb). The eRNA transcribed from this region was induced by AM580 stimulation, but 1,25-vitD alone was ineffective and tended to reduce the effects of AM580 in the combined stimulation. The eRNA transcribed from the fourth binding region (VDR-only) was annotated with *CAMP* (TSS - 0.6 kb). The eRNA transcribed from this binding region was induced by 1,25-vitD stimulation, but AM580 alone was ineffective, and no cooperation was observed (Fig. 4F). Notably, the patterns of eRNA induction were very similar to the patterns of MED1 occupancy in these regions (Fig. 4F).

### Common binding regions of RARα and VDR are often localized in accessible chromatin regions

Signal-regulated TFs (SRTFs) have a limited ability to target DNA motifs in closed chromatin. Therefore, the repertoire of available open regulatory elements (typically established by lineage-determining TFs (LDTFs)) largely defines their binding landscape [87]. However, to a lesser extent, SRTFs bind to genomic regions (called “latent” or “*de novo*” enhancers), which were closed before stimulation, leading to chromatin openness, binding of other TFs, and the acquisition of histone modifications associated with enhancers [87–89]. We previously demonstrated that genomic regions occupied by two or three members of the IRF family were typically more accessible before stimulation than regions occupied by a single IRF [54]. We aimed to investigate whether the common binding regions of RARα and VDR are more accessible compared to regions exclusively occupied by either receptor. Previous studies have clarified the relationship between VDR binding and chromatin accessibility over both short and extended durations, including in human myeloid cells [90–94]. In contrast, studies on RARs have primarily utilized longer treatment durations (9–72 h) to explore the impact of RAR ligands on chromatin accessibility [95–97]. Therefore, we performed ATAC-seq in vehicle- and AM580-treated cells treated for 1 hour with ligand, aligning with the timeframes employed in the RARα and VDR ChIP-seq analyses.

**Figure 5.**
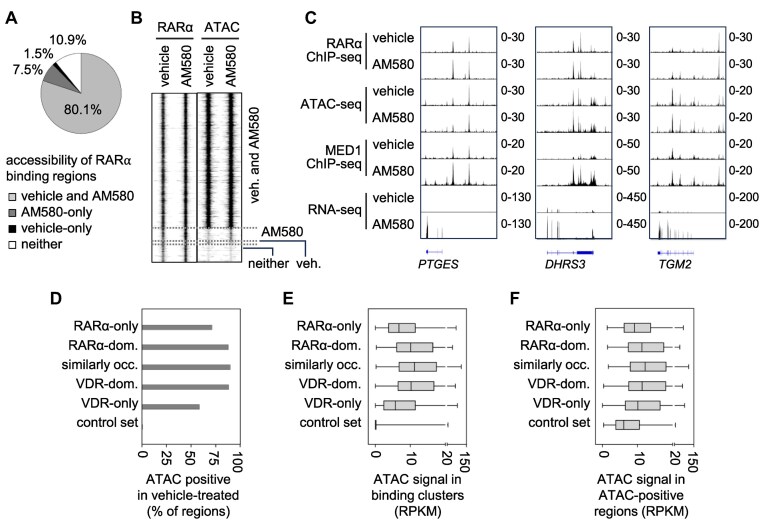
Chromatin accessibility of RARα and VDR binding regions. (**A**) Chromatin accessibility of RARα binding regions determined by ATAC-seq in PMA-THP-1 cells stimulated with vehicle or AM580 for 1 h. The proportions of RARα binding regions that were accessible in vehicle-, and/or AM580-stimulated cells or in neither of these conditions. (**B**) An RD plot showing the RARα ChIP-seq and ATAC-seq signals in 2 kb windows. (**C**) IGV snapshots showing representative genes upregulated by AM580. The tracks display RARα ChIP-seq, ATAC-seq, MED1 ChIP-seq and RNA-seq data (**D**) The percentage of binding regions overlapping with ATAC-positive regions. (E-F) Box-and-whisker plots indicating the ATAC signals in all regions (**E**) and in ATAC-positive regions of each binding cluster (**F**) in cells treated with vehicle.

Most (80.1%) RARα binding regions were accessible in both vehicle- and AM580-stimulated cells (Fig. 5 A and B). Smaller proportions of RARα cistrome were accessible exclusively in AM580-stimulated cells (7.5%) or vehicle-stimulated cells (1.5%). The RARα binding regions that were not accessible in either condition (10.9%) typically exhibited low RARα occupancy levels. Although 18.9% ATAC-seq peaks in AM580-stimulated cells were not detected in vehicle-treated cells (Supplementary Fig. S6A), most of these AM580-only ATAC-seq peaks exhibited low ATAC-seq signals (Supplementary Fig. S6B). As a consequence, only a few (n = 16) regions exhibited significantly different ATAC-seq signals between vehicle- and AM580-treated samples (Supplementary Fig. S6C). We observed that RARα occupancy and chromatin openness did not significantly change in the case of upregulated genes in response to AM580, despite the observed MED1 occupancy being induced upon stimulation (exemplified by *PTGES*, *DHRS3*, and *TGM2* in Fig. 5C). Previous studies reported that RAR ligands (partly likely in an indirect manner) are capable of markedly altering chromatin accessibility [95–97]. The difference observed between our results and previous findings is likely attributable to the differing treatment durations (9–72 h in earlier studies versus 1 h in our study).

In the second analysis of chromatin accessibility, we compared the openness of the five binding clusters in vehicle-treated samples. We found that the fraction of binding clusters overlapping with ATAC-seq positive regions was higher in common binding regions than in RARα-only or VDR-only clusters (Fig. 5D). Additionally, ATAC-seq signal values were higher in common regions when analyzing the entire clusters or regions which overlapped with ATAC-seq peaks (ATAC-positive regions) across different binding clusters (Fig. 5E-F). Thus, we concluded that common binding regions represent more readily accessible genomic sites for these NRs.

### Putative RAREs and VDREs in the RARα and VDR cistromes and binding clusters

We performed *de novo* motif discovery and mapped PWMs in the different peak sets. Analysis of *de novo* motif discovery on the top 1 000 RARα and VDR peaks identified DR5 and DR2 motifs in 14.3% and 14.0% of RARα ChIP-seq peaks, respectively, and the DR3 motif in 54.0% of VDR ChIP-seq peaks (Supplementary Fig. S7). This analysis also revealed additional motifs that are recognized by LDTFs of macrophages, which play critical roles in regulating target genes and establishing enhancers that can be occupied by SRTFs [59, 87, 98, 99].

**Figure 6.**
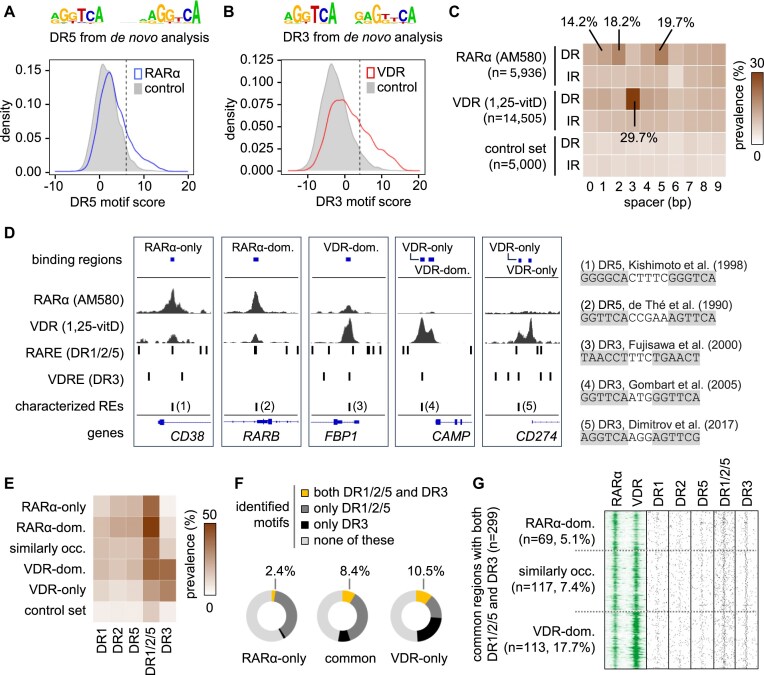
Putative RAREs and VDREs in the RARα and VDR binding regions. (**A-B**) Two density plots showing the distribution of DR5 and DR3 motif scores in RARα binding regions in PMA-THP-1 stimulated with AM580 and VDR binding regions in PMA-THP-1 stimulated with 1,25-vitD. Both plots show the distribution of motif scores in randomly selected size-matched genomic sequences (control, n = 5 000 regions). Score thresholds (dotted lines) were defined as the score giving 5% positive matches in control sequences. The PWMs were obtained from de novo motif analyses of RARα and VDR datasets. (**C**) The prevalence of DRs and inverted repeats (IR) with a spacer length spanning from 0 to 9 nucleotide in entire RARα and VDR cistromes and a control set are presented as a heatmap. (**D**) IGV snapshots showing representative regions with ChIP-seq peaks and response elements. The tracks show the type and position of identified binding regions, RARα and VDR ChIP-seq peaks, the position of retinoic acid response elements (RARE) and vitamin D response elements (VDRE) as determined by our motif enrichment strategy, and the position of elements characterized in previous studies. The scales were determined separately for each of the five regions. Within each region, the same scales were used for both RARα and VDR data. (**E**) A heat map showing the percentage of binding clusters containing DR1, DR2, DR5, DR1/2/5, and DR3 motifs. (**F**) The proportion of RARα-only and VDR-only clusters, as well as common binding regions containing both DR1/2/5 and DR3, only DR1/2/5, only DR3, or none of these. (**G**) ChIP-seq signals and the position of DNA motifs in common binding regions of RARα and VDR containing both DR1/2/5 and DR3.

Motif mapping with PWMs allows direct comparisons of motif prevalence. PWMs of DR3 and DR5 were obtained from databases and refined by *de novo* motif discovery analyses. PWMs of other motifs were generated by modifying the spacer bases of the PWM of DR5 and changing the orientation of the half-sites. Score thresholds for PWMs of DR3 and DR5 were calculated based on control sets containing randomly selected size-matched sequences (for details see Materials and Methods). A score threshold was defined as the score giving 5% of positive matches in the control set (Fig. 6A and B). The relation of thresholds and the percentages of regions containing the motif is shown in Supplementary Fig. S8A. The putative RAREs and VDREs in the cistromes of RARα (AM580-stimulated cells) and VDR (1,25-vitD-stimulated cells) and five binding clusters were investigated in three analyses.

In the first analysis, the entire RARα and VDR cistromes were investigated. The most enriched RARE in the RARα cistrome was DR5 (19.7%) followed by DR2 (18.2%) and DR1 (14.2%) (Fig. 6A, C and Supplementary Fig. S8B). As expected, the most enriched motif in the VDR cistrome was DR3 (29.7%), while other motifs were detected in lower frequencies (Fig. 6B and C and Supplementary Fig. S8B). Notably, the prevalence of DR5 in the RARα cistrome was only 1.5 times higher than in the VDR cistrome (13.3%) (Supplementary Fig. S8B). The binding regions for any of the three most frequent RAREs (DR1, DR2, or DR5, referred to as DR1/2/5) were also determined. In the RARα cistrome and the control set, 42.6% and 15.8% of the regions contained DR1/2/5, respectively (Supplementary Fig. S8B). Only DR1, DR2, and DR5 (DR1/2/5) were included as putative RAREs in further analyses because adding other motifs would further increase the background (as demonstrated by the control set). Notably, motif mapping detected several previously characterized response elements for example the ones associated with *CD38, RARB*, *FBP1*, *CAMP*, and *CD274* [69, 72, 77, 100, 101]. These motifs were found close to the summit of the RARα and/or VDR peaks in our study (Fig. 6D). Although the well-characterized regulatory region of *RARB* [30, 100] was classified as a RARα-dominant region with DR5 motif in our system, the gene expression did not change by any ligand. This along with the observation that most of the binding regions were annotated with genes that were not regulated (Fig. 3B) highlights that the binding of RARα and VDR per se is not sufficient for gene expression changes.

In the second analysis, the prevalences of various RAREs (DR1, DR2, DR5, and DR1/2/5) and DR3 motifs in the five binding clusters were analyzed (Fig. 6E). As expected, in the “RARα-only” and “RARα-dominant” clusters high RARE and low DR3 frequencies were detected. In the “similarly-occupied” cluster, both types of response elements were enriched, but DR1/2/5 were 3.1 times more frequent than DR3 (40.6% and 13.1%, respectively). Unexpectedly, in the “VDR-dominant” and “VDR-only” clusters not only DR3 but RAREs were also enriched. The relatively high frequency of DR1/2/5 in the VDR-only cluster (26.2%) raised the question of why RARα did not occupy these regions. Based on the analysis of ATAC-seq data and motif scores, we concluded that these regions may not be favored by RARα because the DR2 and DR5 scores were typically lower than in other clusters (Supplementary Fig. S8C) and these binding regions were localized to chromatin regions with low accessibility (Fig. 5D-F).

In the third analysis, binding regions containing both RARE (DR1/2/5) and VDRE (DR3) were analyzed (Fig. 6F-G). This analysis revealed that the co-occurrence of the two motif types was low in the RARα-only cluster (2.4%) but higher in the common clusters (average: 8.4%) and VDR-only (10.5%) cluster. Among the three clusters of common regions, the prevalence of regions containing the two motif types was the highest in the VDR-dominant cluster (17.7%, 113 out of 637 regions) (Fig. 6G). Our results indicated that a large majority of common clusters contained only one type (44.4%) or none (47.2%) of DR1/2/5 and DR3 motifs (Fig. 6F, the middle chart). Overall, these analyses suggested that only a small portion of RARα and VDR co-binding was mediated via “strong” co-occurring canonical motifs.

### Common binding regions of RARα and VDR contain RARE and VDRE in various configurations

**Figure 7.**
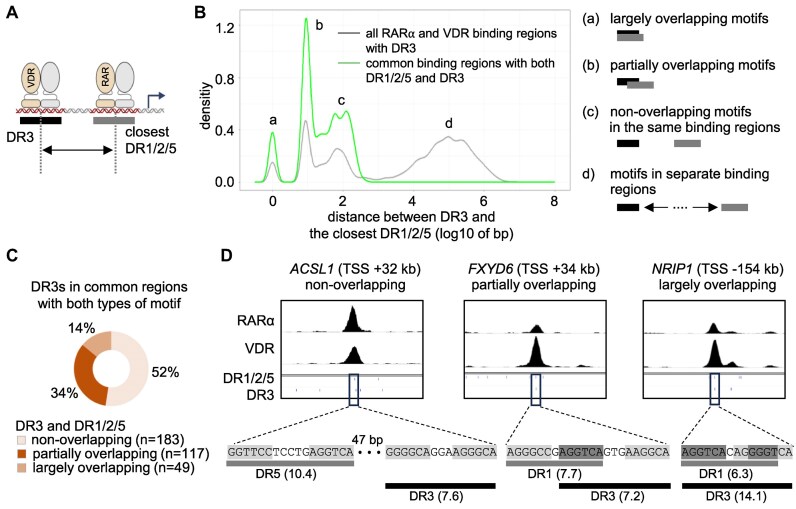
Composition of RAREs and VDREs in the common binding regions of RARα and VDR (**A**) A scheme showing the position of bases used for the calculation of distances between the DNA motifs. (**B**) The distances between DR3 and the closest DR1/2/5 are shown on a density plot. The distances were calculated for DR3 motifs identified in all RARα and VDR binding regions (grey) or the common binding regions of RARα and VDR (green) containing both DR1/2/5 and DR3. The four indicated populations (a–d) represent different motif compositions. (**C**) The classification of DR3 motifs that localize in the common binding regions of RARα and VDR containing both DR1/2/5 and DR3. (**D**) IGV snapshots showing representative genomic loci (peak summits ± 3 kb) containing DR3 and DR1/2/5 in different configurations. The numbers in parentheses indicate the motif scores.

We determined the configurations of co-occurring DR1/2/5 and DR3 motifs in the common binding regions. The distances were calculated between the middle base of DR3 and the middle base of the closest DR1, DR2, or DR5 (Fig. 7A). Based on the number of bases in DR3 that were also parts of DR1/2/5, the configurations of motifs were classified as largely overlapping (7 or more bases), partially overlapping (1–6 bases), or non-overlapping (none) motifs. All three types of configurations were detected when the distances were analyzed in the common binding regions that contained both DR1/2/5 and DR3 motifs (Fig. 7B, green line). When we analyzed the distribution of distances in all RARα and VDR binding regions containing DR3 motifs (Fig. 7B, grey line), similar three populations were identified together with a population in which the closest DR1/2/5 was localized in an entirely separate peak than the DR3 containing peak. We performed more detailed systematic analyses to determine the frequency of configurations in common regions containing both types of motifs (DR1/2/5 and DR3). These analyses revealed that the most frequent configuration was the non-overlapping motif (52%), followed by partially overlapping (34%), and largely overlapping (14%) motif configuration (Fig. 7C). Examples of the three motif configurations are shown in Fig. 7D.

### Overlap of nuclear receptor cistromes and co-occurrence of motifs in other studies

**Figure 8.**
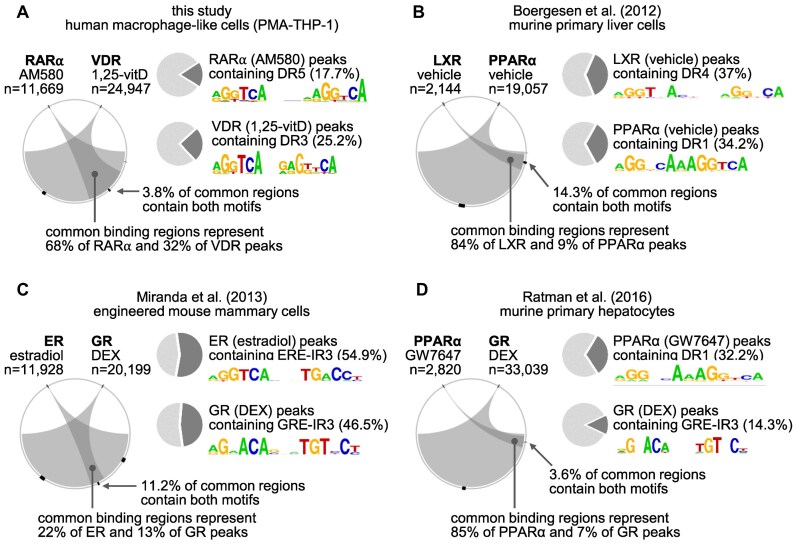
Overlap of nuclear receptor cistromes and co-occurrences of DNA motifs. (**A**) Our datasets and (**B–D**) publicly available ChIP-seq datasets were re-analyzed. Circos plots show the overlap of nuclear receptor cistromes. The most enriched motifs were determined for each NR, and their logo with the prevalences (in pie charts) in the entire cistromes are indicated. Co-occurrences of motif pairs were calculated for three sets of genomic regions (two receptor-specific and one common). The proportions for all sets are indicated by block arcs, but the accurate percentages are specified only for the common sets.

We wanted to explore the extent of NR cistrome overlaps and the co-occurrence of motifs in our study and other studies. A series of previous studies identified the binding regions of two NRs with different motif preferences (Supplementary Table S5) [42, 44, 45, 102, 103]. Many of these studies documented that the cistromes of NR pairs largely overlapped in various cell types [42, 44, 45, 103]. The authors of these studies used different peak calling algorithms and motif discovery strategies; thus, the results could not be directly compared. Therefore, we decided to reanalyze ChIP-seq datasets of selected studies [44, 45, 102] along with our datasets using a standardized workflow. (Because of the lack of replicates in some studies, we could not use our original workflow.) In each pairwise NR comparison, we identified binding regions and calculated the overlap between the NR cistromes, the prevalence of the most enriched motifs, and the co-occurrence of motifs (Fig. 8).

The ratios of larger over smaller cistrome were 2.1 for VDR versus RARα, 1.7 for ERα versus GR, 8.9 for LXR versus PPARα, and 11.7 for PPARα versus GR (Fig. 8A–D). The fraction of overlapping regions in the smaller cistrome ranged from 22% (ERα cistrome in the ERα versus GR comparison, Fig. 8C) to 85% (PPARα cistrome in the PPARα versus GR comparison, Fig. 8D). (The size of the RARα and VDR cistromes and the overlap were slightly different from the results provided earlier in this study (Fig. 1A) because of the alternative workflow.)

We determined the PWM of the most enriched motif for each NR as described in the “Materials and methods” section. Using control sets, the thresholds were defined for the motifs (as described previously), and the motif prevalences were determined (Fig. 8A–D). Among the putative RAREs, only the occurrence of DR5 was re-analyzed in the RARα cistrome for the sake of simplicity. Because of the different peak identification strategy, the prevalences of motifs in the RARα and VDR datasets were lower than calculated earlier (see percentages in Fig. 6C versus Fig. 8A). We found that the standardized workflow resulted in very different motif prevalences ranging from 14.3% (GR peaks containing GRE-IR3) to 54.9% (ER peaks containing ERE-IR3). When co-occurrences of motifs were analyzed in the common binding regions, low frequencies were detected in all studies (ranging from 3.6% to 14.3% of common peaks, Fig. 8A–D). Even in the ER vs. GR comparison, where the prevalence of motifs was high in entire cistromes, the co-occurrence of the two motifs was detected only in 11.2% of common binding regions (Fig. 8C). Collectively, the re-analyses of datasets indicated that the extensive sharing of binding regions without a high frequency of motif co-occurrence is a general phenomenon among NRs.

## Discussion

### Common binding regions are at the cross-roads of retinoid and vitamin D pathways

Transcriptional programs regulated by NRs represent overlapping gene sets. A series of molecular mechanisms were proposed to explain why a certain gene is regulated by agonists for two or more NRs (reviewed in [104, 105]). (i) Two NRs may regulate target genes via DNA binding at the same regulatory elements; (ii) NRs can regulate the same gene via DNA binding at separate regulatory elements; (iii) one NR may regulate its target gene directly while the other one indirectly regulates the gene via tethering [105, 106]; and (iv) one NR regulates the expression of another NR or enzyme(s) involved in the production of endogenous agonists for another NR.

We observed that the common binding regions were more frequently annotated with genes that were commonly upregulated compared to the RARα-only and VDR-only clusters (Fig. 3B). The correlation analysis demonstrated an overrepresentation of common peaks in the TSS ± 25 kb of genes upregulated by both ligands (Fig. 3D). Moreover, a large proportion of common binding regions were localized in accessible chromatin (Fig. 5D), and in many cases, MED1 was recruited to these regions in a ligand-responsive manner (Fig. 4B). Our analyses collectively suggest that the first mechanism, namely, gene regulation by two NRs via DNA binding at the same regulatory elements, plays an active and important role in the convergence of retinoid and vitamin D signaling pathways.

### DNA binding by RARα and VDR is not always coupled with ligand-induced cofactor recruitment and gene regulation

Although our findings suggest an active and important role for common binding regions in the convergence of the two pathways, many of these common regions (as well as other types of RARα and VDR binding regions) are likely not involved in transcriptional regulation mediated by cofactor recruitment. We found that most RARα and VDR binding regions were annotated with genes that were not regulated by the ligands (Fig. 3B). Moreover, we observed that the MED1 signal at a large proportion of RARα and VDR binding regions was not induced by the ligands (Fig. 4A and B). In the case of common binding regions, we observed all three possibilities: MED1 recruitment was either induced by both ligands, only by one ligand, or by none of the ligands. For example, both ligands were able to induce MED1 signals at common regions and increase mRNA levels, as exemplified by *FBP1* and *HBEGF* (Figs. 2E, 3E, and 4E). In contrast, the common binding region associated with *PTGES* was occupied by both receptors upon ligand treatment, but only AM580 could induce the MED1 signal (Figs. 2E, 3E, and 4E). These data, consistent with studies on NRs and various TFs, suggest that only a subset of binding events is coupled with ligand-induced cofactor recruitment and gene regulation.

Several determinants have been previously characterized that influence the ability of DNA-bound TFs to recruit cofactors and regulate gene expression. (i) DNA-bound TFs cannot function as transcriptional regulators without proper contact with the promoter. Physical interactions between regulatory elements primarily occur through loops facilitated by chromosomal architecture and are essential for achieving gene regulation [107, 108]. (ii) TF binding affinity to a binding region can be associated with gene-activating capacity. It has been suggested that stronger TF binding affinity to the DNA is coupled with a higher gene-activating capacity [109, 110]. (iii) Core DNA sequences, flanking regions, and motif architecture allosterically modulate TF structure and encode distinct gene regulatory effects [111–113]. (iv) Because TFs work in collaboration with other TFs and non-DNA-binding coregulators, the regulatory activity of a given TF is influenced by its co-binding TFs [85, 114–116 ]. (v) TF activity also depends on the binding-site strand, position, DNA helical face and chromatin context [117, 118]. These determinants, in a combinatorial and interacting fashion, define whether a given TF (in our case, RARα and VDR) at a specific regulatory element can recruit cofactors and regulate gene expression.

### A large proportion of RARα and VDR binding regions does not contain “strong” response elements, therefore the prevalence of motif co-occurrence is low in the common regions

Less than 10% of common binding regions of RARα and VDR contain both types of “strong" motifs (DR1/2/5 and DR3) (Fig. 6F and G), consistent with a previous study on LXR and PPARα [45] and the re-analyses of publically available ChIP-seq datasets (Fig. 8). Our results suggest that the frequency of co-occurring motifs is low because large portions of RARα and VDR binding regions do not contain “strong” response elements.

No ultimate method is available for mapping motifs and separating “strong” and “weak” (degenerate) motifs. The prevalence of a given motif in a TF cistrome can be primarily evaluated by three methods: mapping PWMs, *de novo* motif discovery, and searching for specific DNA sequences. The three strategies have advantages and limitations, as described in our previous study [54]. In this study, we used a relatively stringent version of the first method in which the threshold yielded 5% of positive matches in the control set. Stringency should be relatively high to reduce the background and the likelihood of false positive results.

A series of observations support the suitability of our motif mapping strategy for the well-balanced identification of putative RARE and VDRE in the cistromes and binding clusters. First, our motif mapping strategy detected several previously characterized response elements associated with *RARB*, *FBP1*, *CD274*, *CAMP*, and *CD38* [69, 72, 77, 100, 101] (Fig. 6D). Second, the pattern of motif prevalences in the RARα and VDR cistromes (Fig. 6C) resembles the binding preferences of these NRs, as determined by high-throughput SELEX [34]. Third, the ERE-IR3 was detected in ∼55% (Fig. 8C) of the ER cistromes in engineered mouse mammary cells [102], implying that our mapping method efficiently detected most motifs in a ChIP-seq dataset. Last, the authors investigating RAR and VDR datasets also reported that more than half of the cistromes do not contain cognate motifs. For example, using different methods, the DR5 was identified in ∼6% and ∼40% of RAR cistromes in mouse embryonic stem cells and embryonal carcinoma cells, respectively [32, 33]. Notably, DR5 was not the most enriched RARE in these studies. In our study, DR5 was the most enriched RARE and was detected in 19.7% of the RARα binding regions in PMA-THP-1 cells (Fig. 6C). RAR-RXR binds to DR5 and other response elements [32–34, 119]; therefore, we included two other motifs (DR1, DR2) as putative RAREs. Similar to our study, the frequency of DR1/2/5 was determined in the RAR cistrome in mouse F9 embryonal carcinoma cells [33]. The frequency was lower in our study (Supplementary Fig. S8B) than in embryonal carcinoma cells (40.2% vs. 74.3%), probably because they used a less stringent motif mapping strategy and an anti-panRAR antibody. The prevalence of DR3 motif, the most enriched VDRE, was 29.7% in the VDR cistrome in our study (Fig. 6C). DR3 was identified in ∼42% and ∼48% of VDR cistromes in LPS-treated THP-1 and undifferentiated THP-1, respectively [35, 36]. In 1,25-vitD stimulated monocyte-derived dendritic cells, *de novo* motif discovery analysis showed that ∼37% of VDR binding regions contained the DR3 motif [37]. These studies illustrate the common observation that a significant proportion of the RARs and VDR cistromes do not contain DR5 and DR3 motifs, respectively.

Different models can explain the lack of a “strong” motif in a large proportion of NR cistromes (reviewed in [105, 120]). (i) NRs can bind to “weak” or degenerate motifs. The chromatin accessibility largely determines the binding events; therefore, the same “weak” motifs may represent high and low-affinity sites in open and closed chromatin regions, respectively. (ii) Many TFs, including NRs, can bind and act through a diverse set of motifs [34, 120]. Although we considered DR1/2/5 as RAREs, we cannot exclude the possibility of RARα-RXR binding to other motifs. Indeed, other motifs were enriched (though with low frequencies) in the RARα cistrome in our analysis (Fig. 6C) and previous studies on RAR-RXR. Similarly, various studies demonstrated that VDR-RXR binds to other response elements, such as DR2, DR4, and even DR5 (in a transcriptionally inactive form) [30, 34, 121]. RARα and VDR can bind to a broader and more varied set of response elements than the putative response elements in this study. (iii) Protein-binding microarrays indicate that NR binding can occur in half-site modes [119]. (iv) NRs can bind DNA indirectly via protein-protein interactions, allowing NRs to bind other NRs or other TFs (e.g. trans-repression, tethering) [105, 120]. Most likely, many of these mechanisms are responsible for RARα and VDR binding to regions that do not contain “strong” motifs, but their relative contribution cannot be evaluated without high-resolution TF mapping.

### Non-overlapping and shared DNA sequences in common regions of RARα and VDR

In the absence of high-resolution TF mapping, the interaction sites between TFs and DNA cannot be accurately defined. To our knowledge, ChIP-exo (a method for high-resolution TF mapping [122]) has not been used for analyzing shared binding regions of NRs, and the adoption of this method was out of the scope of this study. However, mapping the cognate DNA motifs could help to predict whether they predominantly bind separate DNA sequences or share binding sites. By calculating the distances between DR3 and the closest DR1/2/5, we could determine the proportion of three configurations of motifs in the common binding regions. We found that approximately half (52%) of DR3 motifs did not overlap with DR1/2/5, while other DR3 motifs largely (14%) or partially (34%) overlapped (Fig. 7C). The main difference between the two types of overlapping motifs is that only one half-site was shared by the two heterodimers in the partially overlapping motifs.

Boergesen *et al.* also investigated shared DNA motifs in murine primary liver cells [45], using a different bioinformatics approach (comparison of distances between peak centers). They demonstrated that the average distances between LXR and PPARα peak centers were very close to the average distances between the peak centers of LXR-RXR and PPARα-RXR and the distribution patterns were highly similar. Based on these results, they concluded that LXR-RXR and PPARα-RXR were predominantly binding to the same or overlapping degenerate response elements at these sites. Moreover, the analyses of the binding regions for other NRs (HNF4α, FXR, and NR1D1) indicated the existence of NR “hot spots” in the liver to which multiple NRs bind with considerable sharing of degenerate DR elements [45]. Although the prevalences of non-overlapping motifs in our study are different from the results of Boergesen *et al.*, both studies highlight the importance of shared motifs in the convergence of NR pathways.

### Cooperation and competition of NRs at the common regulatory elements that contain both types of motifs

The observation that various NR dimers may bind to shared motifs in response to combined ligand treatment, raises the question of whether NR dimers compete for binding to these shared sites or co-occupy without competition. Fluorescent protein labeling coupled with live cell imaging was employed to address this question. Different live-cell imaging experiments revealed the highly dynamic binding behavior of TFs in live cells, suggesting that TFs typically interact transiently with their response elements [123]. Using GFP-labeled GR and mCherry-labeled ER pBox (modified ER that binds the same response elements as GR) and fluorescence microscopy and other methods, Voss [124] demonstrated that the GR and ER pBox do not significantly compete for binding to the same response element. Moreover, GR “assissted” binding of the ER pBox at a large subset of binding elements, even though they bound to the same site. Collectively, these results suggest that two activated NRs can bind shared response elements without competition.

We demonstrated that common binding regions are involved in the cooperation between retinoid and vitamin D pathways; however, combined stimulation was not always cooperative, even in common binding regions (Fig. 4). The functional outcome of NR co-activation in common regions is context-dependent, and most likely the outcome is determined by the chromatin status, binding of other TFs, the affinity of the individual motifs, and the configurations of co-occurring DNA motifs. Cooperative NR activity at a common region is most likely achieved via more efficient cofactor recruitment, chromatin remodeling, histone modification, and increased activation of the basal transcriptional apparatus. In summary, growing evidence about the overlapping NR binding regions and transcriptional programs has emerged with the help of high-throughput methods. Our study sheds light on how NRs with different response element repertoires regulate largely overlapping sets of regulatory regions and genes.

## Supplementary Material

gkaf230_Supplemental_Files

## Data Availability

The data underlying this article are available in NCBI Gene Expression Omnibus (GEO) at http://www.ncbi.nlm.nih.gov/geo/, and can be accessed with GSE246310.
