# Supplementary material for: Genomic regions occupied by both RARα and VDR are involved in the convergence and cooperation of retinoid and vitamin D signaling pathways
Source: Nucleic Acids Res. 2025 Apr 1;53(6):gkaf230. doi: 10.1093/nar/gkaf230 (PMC11959543; doi:10.1093/nar/gkaf230)
Supplement: gkaf230_Supplemental_Files [file gkaf230_supplemental_files.zip › Mianesaz_supplementary_figures_v26-R2.pdf]

Figure S1

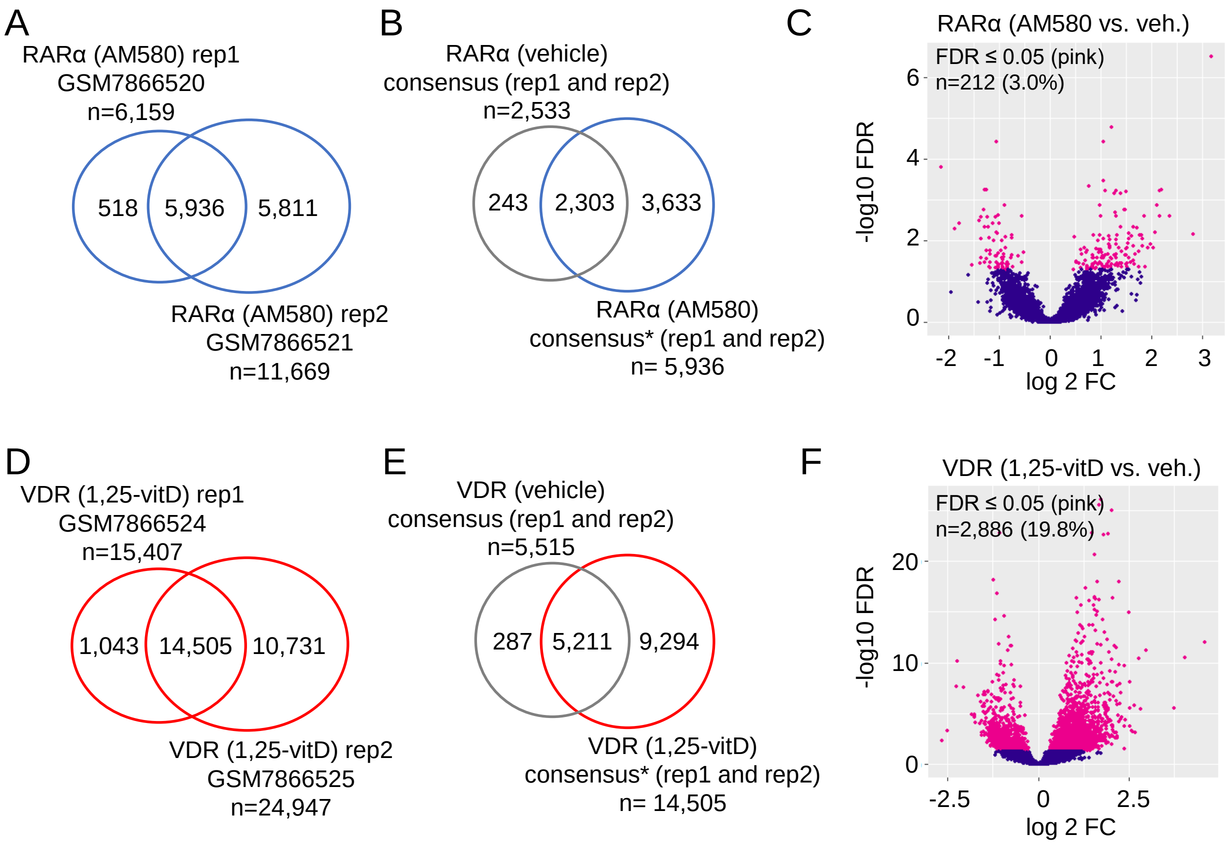

Figure S1. Comparison of RARα and VDR peak sets. (A) The overlap between two sets of RARα peaks (replicates 1 and 2) in PMA-THP-1 cells stimulated with 100 nM AM580 for 1 hour. The consensus peak sets were determined by a specialized method (see Materials and Methods). (B) The overlap of consensus RARα peak sets identified in samples treated with vehicle or AM580. The asterisk indicates the first input file used for intersectBed. (C) Volcano plot displays RARα binding log2 fold change versus -log10 FDR determined by DiffBind. Regions are color-coded based on significance: FDR ≤ 0.05 (pink) and FDR > 0.05 (blue). The number of regions with FDR ≤ 0.05 and the fraction of the consensus RARα peak set (determined by DiffBind) with FDR ≤ 0.05 are indicated in the plot. (D) The overlap between two sets of VDR peaks (replicates 1 and 2) in PMA-THP-1 cells stimulated with 100 nM 1,25-vitD for 1 hour. The consensus peak sets were determined by a specialized method (see Materials and Methods). (E) The overlap of consensus VDR peak sets identified in samples treated with vehicle or 1,25-vitD. The asterisk indicates the first input file used for intersectBed. (F) Volcano plot displays VDR binding log2 fold change versus -log10 FDR determined by DiffBind. Regions are colour-coded based on significance: FDR ≤ 0.05 (pink) and FDR > 0.05 (blue). The number of regions with FDR ≤ 0.05 and the fraction of the consensus VDR peak set (determined by DiffBind) with FDR ≤ 0.05 are indicated in the plot.

Figure S2

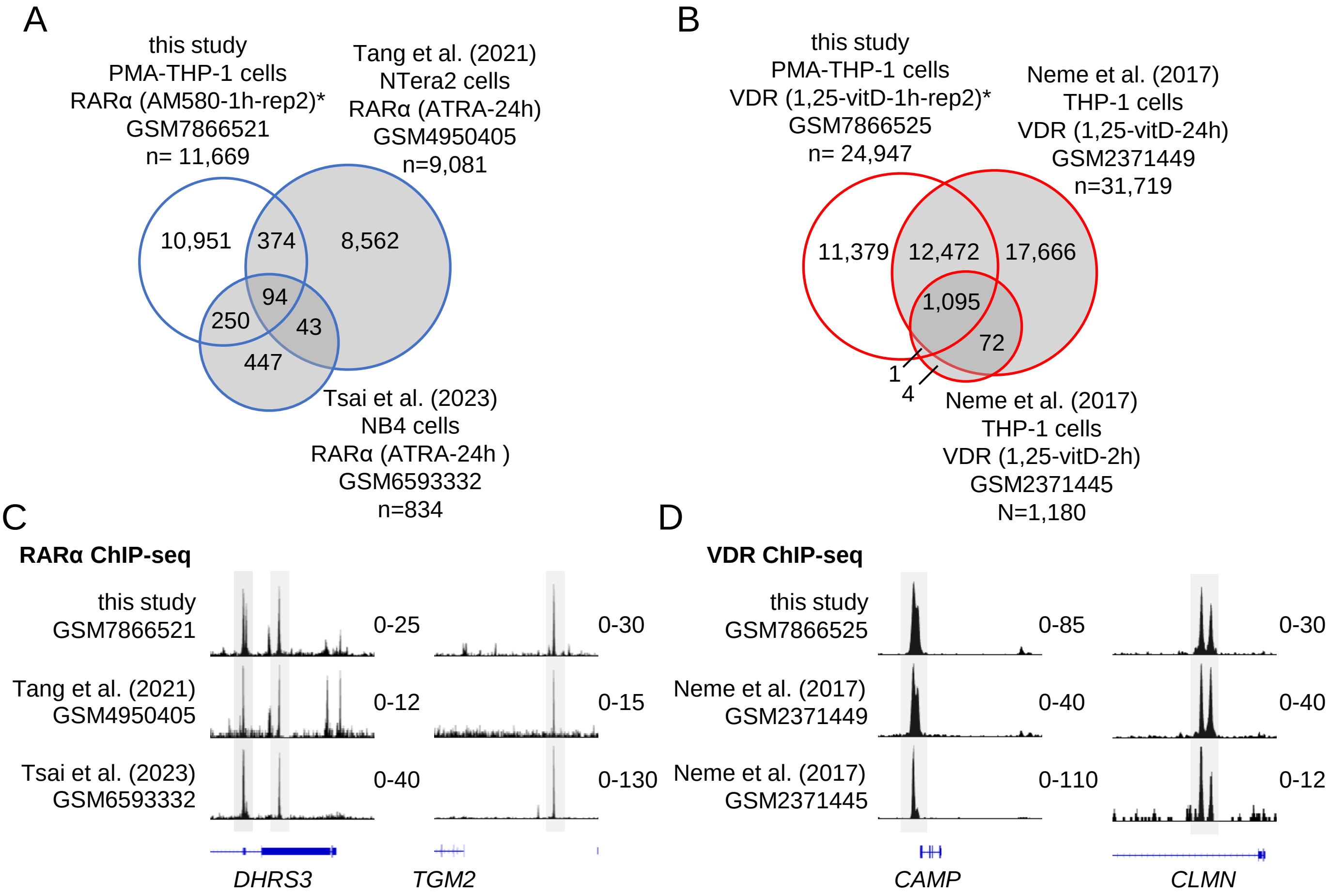

Figure S2. Comparison of RARα and VDR peak sets with other previously published ChIP-seq data sets. (A-B) The overlap of RARα and VDR cistromes determined in our study with other studies (the studies are listed in the References). The asterisk indicates the first input file used for intersectBed. (C-D) Integrative Genomics Viewer (IGV) snapshots showing the RARα and VDR target genes. The cells were treated with ligands as indicated in panels A–B.

Figure S3

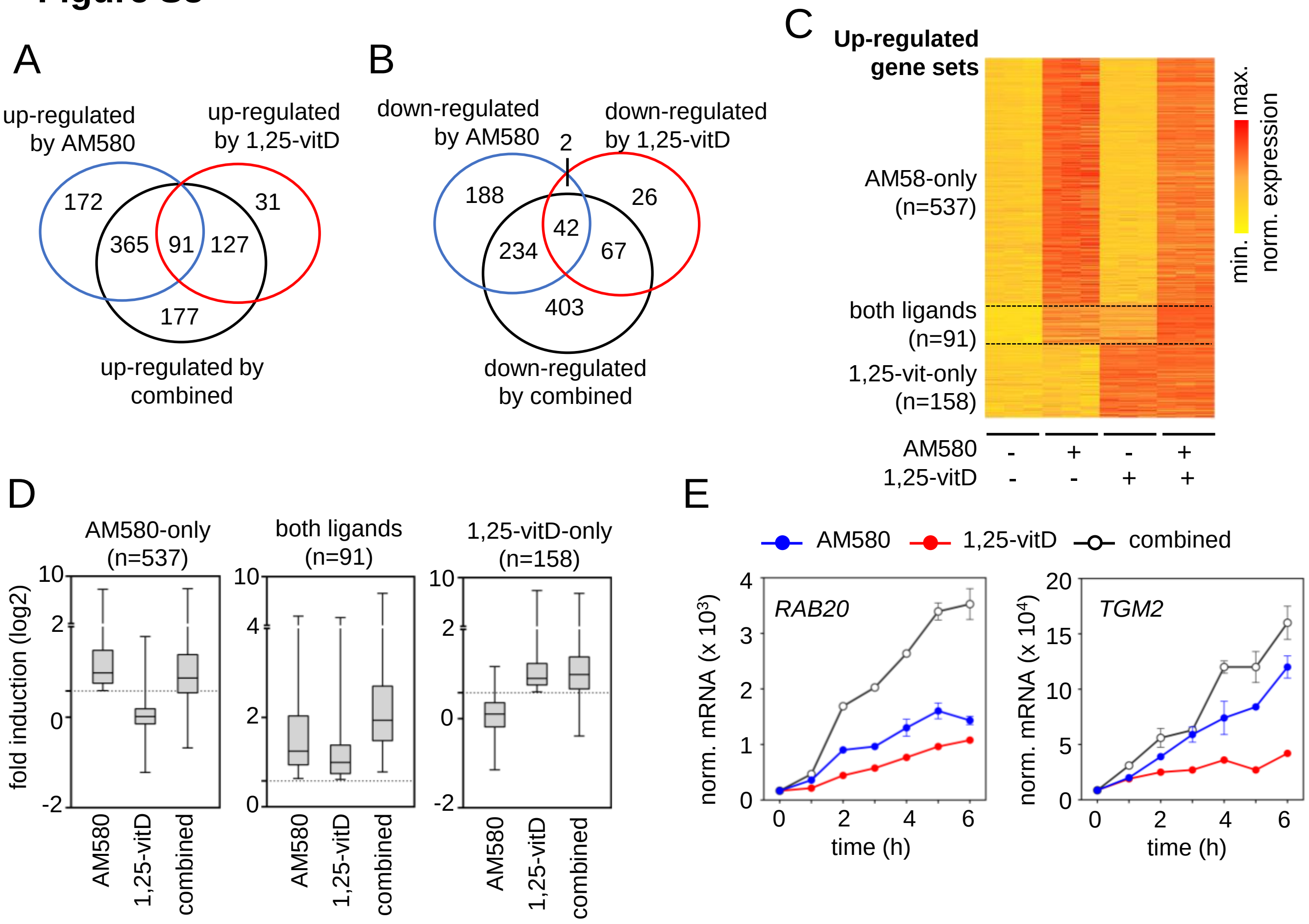

Figure S3. The overlap and the expression pattern of genes regulated in PMA-THP-1 cells treated with vehicle or ligand(s). (A-B) Venn diagrams displaying the overlap between up- or down-regulated genes. PMA-THP-1 cells were treated with vehicle, RARα agonist (AM580), VDR agonist (1,25-vitD), or both ligands (combined) for 6 hours. (C) Heatmap of up-regulated gene sets showing the expression values of three replicates per condition. The heatmap was generated by DisplayR, the values were row normalized (adjusting the values in each row independently). (D) Box-and-whisker plots showing fold changes in three sets of up-regulated genes. (E) Time courses of the mRNA expression of two representative genes measured by RT-qPCR. Expression levels were normalized to *PPIA,* and one representative experiment out of three is shown. Values are expressed as the mean of technical triplicates ± SD of the mean.

Figure S4

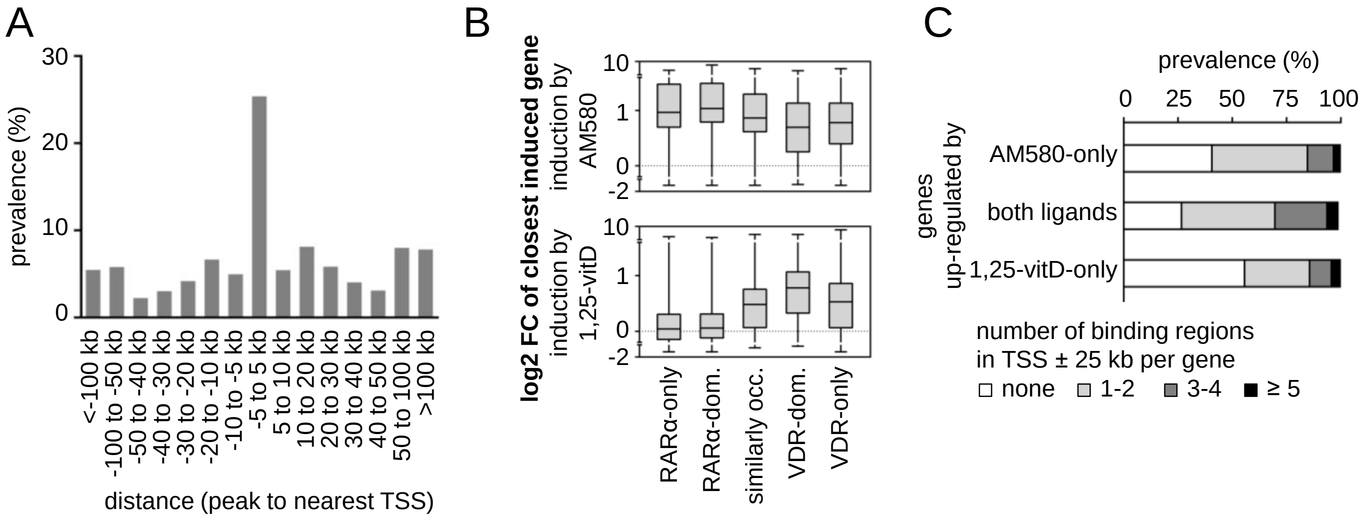

Figure S4. Relationship between receptor binding and gene induction. (A) Distances between RARα and VDR binding regions and the closest annotated transcription start site (TSS). (B) Box-and-whisker plots showing the fold change induced by AM580 (top) and 1,25-vitD (bottom) in different binding clusters. For each binding region, the fold change values were determined for the closest up-regulated gene. (C) A graph indicating the number of the binding regions per gene in the TSS ± 25 kb of genes in three up-regulated gene sets.

Figure S5

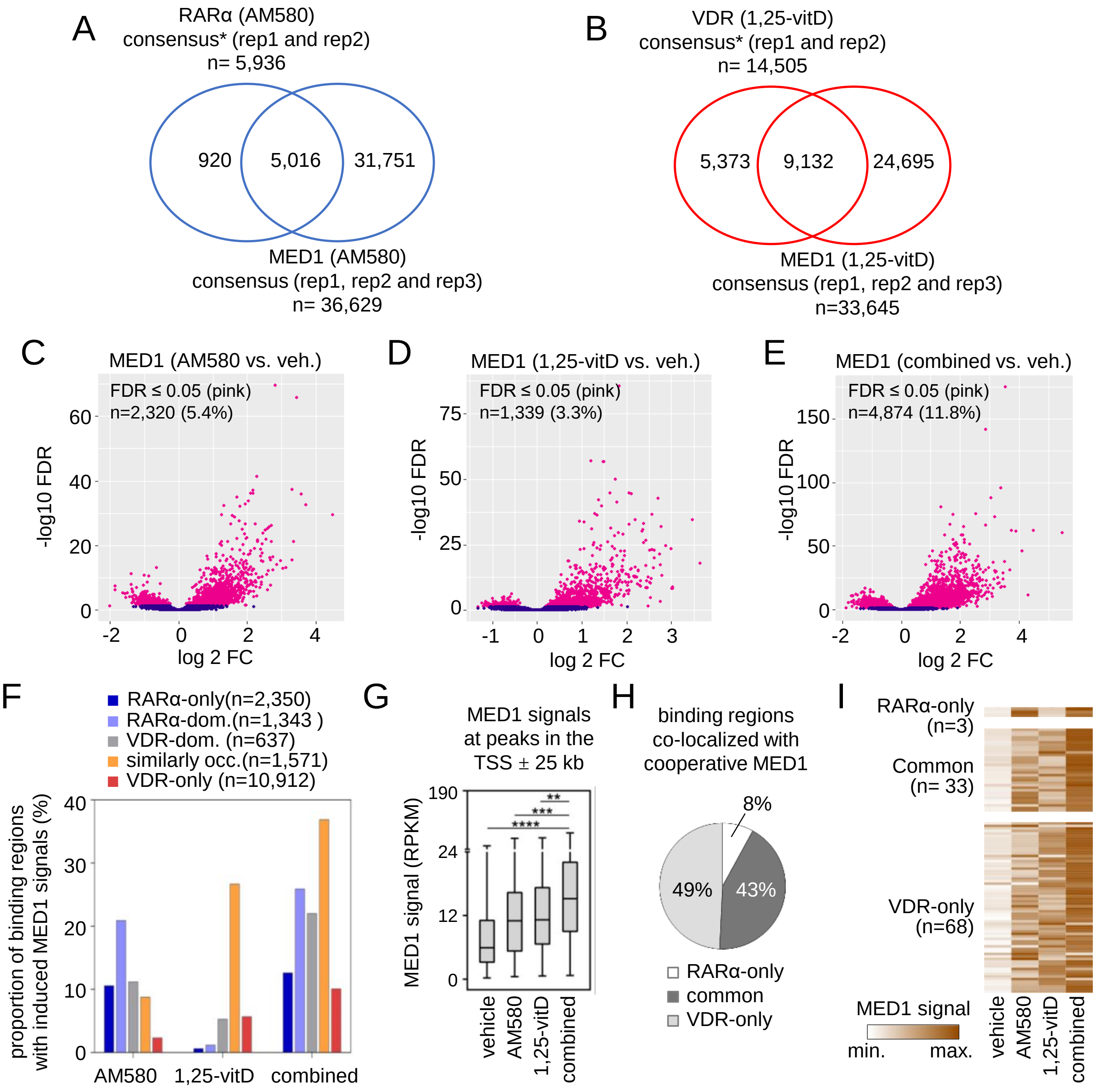

Figure S5. Ligand-induced MED1 occupancy at RARα and VDR binding regions. (A-B) The overlap between RARα and MED1 consensus peak sets in cells treated with AM580 (A), and VDR and MED1 consensus peak sets in cells treated with 1,25-vitD (B). The asterisks indicate the first input file used for intersectBed. (C-E) Volcano plots displaying the MED1 binding log2 fold change versus -log10 FDR calculated by DiffBind in cells treated with ligand(s) versus vehicle. Regions are color-coded based on significance: FDR ≤ 0.05 (pink) and FDR > 0.05 (blue). The number of regions with FDR ≤ 0.05 and the fraction of each consensus MED1 peak set (determined by DiffBind) with FDR ≤ 0.05 are indicated in each plot. (F) The proportion of binding regions with significant induced MED1 signal (DiffBind, FDR ≤ 0.05) upon treatment with AM580, 1,25-vitD or both ligands (combined). (G) A box-and-whisker plot showing the MED1 signals at binding regions (n=104) falling in TSS ± 25 kb of the cooperatively up-regulated gene. The average MED1 RPKM was calculated for three replicates per condition. The significance was determined by ANOVA followed by Dunnett's multiple comparisons test. ****, p-value < 0.0001; ***, p-value=0.0003; **, p-value=0.0040. (H) Pie chart showing the proportions of binding clusters that were co-localized with cooperative MED1, where the signals were significantly higher in the combined treatment than in the more effective single treatment (p ≤ 0.05). (I) Heatmaps displaying MED1 occupancy at binding regions (n=104) falling in TSS ± 25 kb of the cooperatively up-regulated gene set. The average MED1 RPKM was calculated for three replicates per treatment condition. The heatmap was generated by DisplayR, the values were row normalized (adjusting the values in each row independently).

Figure S6

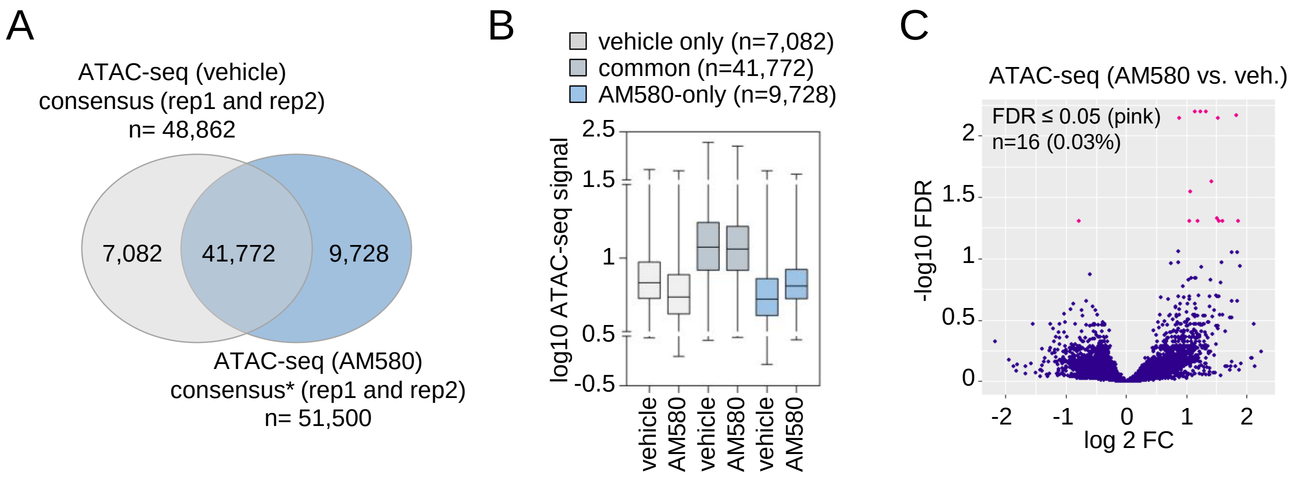

Figure S6. Analysis of accessible chromatin in PMA-THP-1 cells treated with vehicle and AM580. (A) A Venn diagram showing the overlap between ATAC-seq consensus peak sets in cells treated with vehicle or AM580. The asterisk indicates the first input file used for intersectBed. (B) Box-and-whisker plot depicting the ATAC-seq signal intensity across various ATAC-seq peak sets. (C) Volcano plot displays the log2 fold change in ATAC signal versus -log10 FDR calculated by DiffBind. Regions are color-coded based on significance: FDR ≤ 0.05 (pink) and FDR > 0.05 (blue). The number of regions with FDR ≤ 0.05 and the fraction of consensus ATAC-seq peak set (determined by DiffBind) with FDR ≤ 0.05 are indicated in the plot.

Figure S7

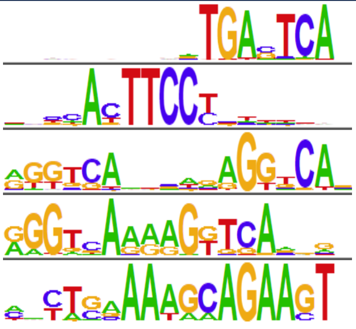

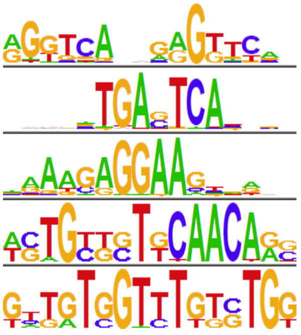

	best match	p-value	% target	% bg
RARα (AM580)	JunB (bZIP)	1e-205	28.40	2.50
	PU.1 (EST)	1e-156	27.00	3.38
	RAR:RXR (DR5)	1e-130	14.30	0.75
	Rarg	1e-74	14.00	1.86
	SPI1	1e-22	1.10	0.01
VDR (1,25-vitD)	VDR (DR3)	1e-654	54.00	1.75
	Atf3 (bZIP)	1e-145	26.60	3.61
	SpiB (ETS)	1e-87	17.80	2.68
	Aef1	1e-20	0.90	0.00
	RUNX1 (Runt)	1e-20	0.90	0.00

Figure S7. The results of HOMER *de novo* motif discovery using the top 1000 peaks of the RARα ChIP-seq from AM580-stimulated cells (top) and VDR ChIP-seq from 1,25-vitD-stimulated cells (bottom). The following parameters were used: motif lengths of 17, 18 bp (RARα) and 15, 16 bp (VDR); region size: 200 bp. bg, background.

Figure S8

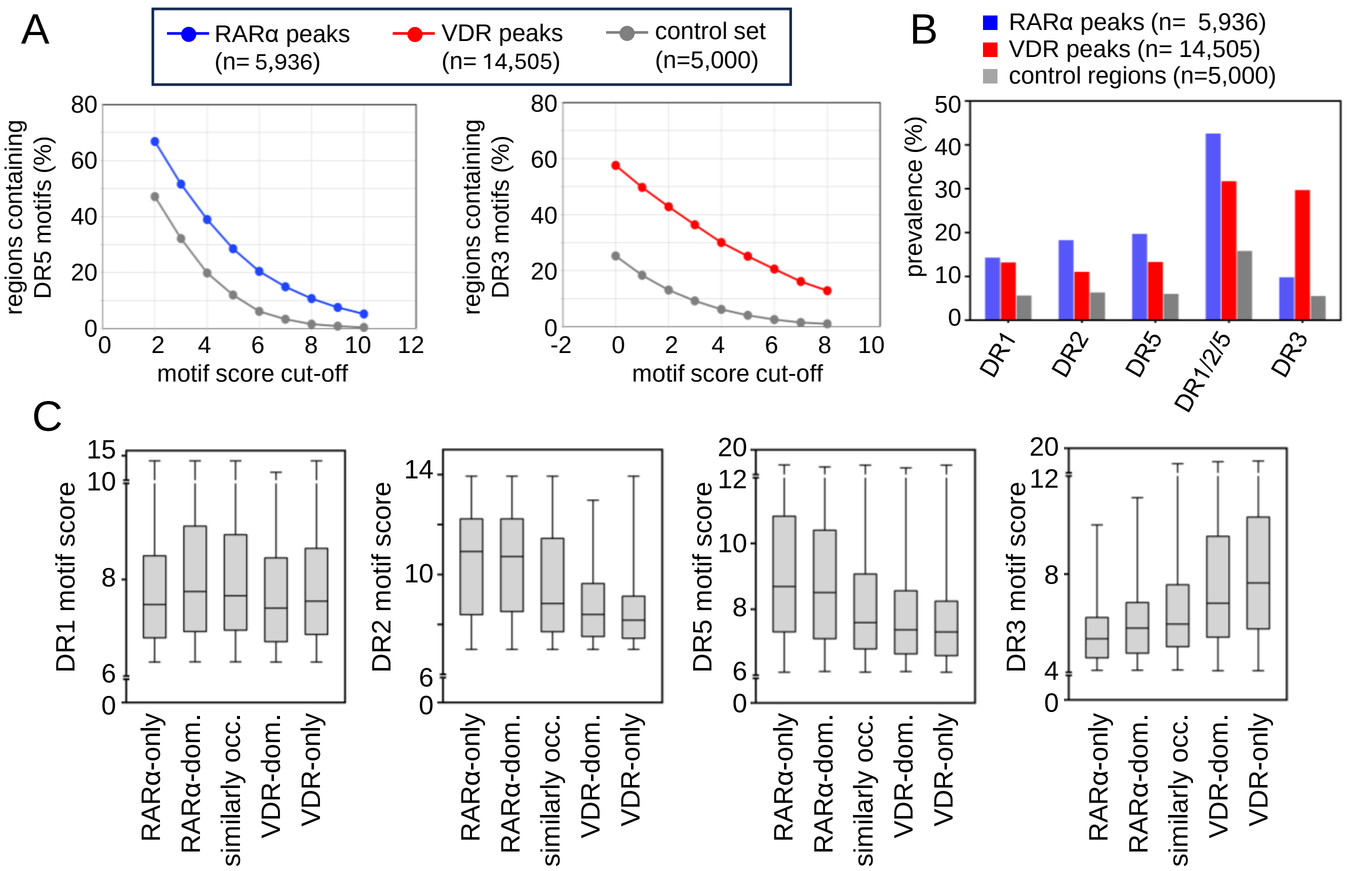

Figure S8. Characterization of DNA motifs in RARα and VDR binding regions. (A) The prevalence of DR5 and DR3 motifs in the entire RARα and VDR cistromes and control set (randomly selected, size-matched genomic sequences) using various motif score cut-offs. (B) Prevalence of DR1, DR2, DR5, DR1/2/5 (any of DR1, DR2, and DR5), and DR3 in the RARα and VDR cistromes and the control set. (C) Box-and-whisker plots showing the highest motif score above the threshold in different binding clusters.
