# Supplementary material for: Genomic regions occupied by both RARα and VDR are involved in the convergence and cooperation of retinoid and vitamin D signaling pathways
Source: Nucleic Acids Res. 2025 Apr 1;53(6):gkaf230. doi: 10.1093/nar/gkaf230 (PMC11959543; doi:10.1093/nar/gkaf230)
Supplement: gkaf230_Supplemental_Files [file gkaf230_supplemental_files.zip › SUPPORTING_INFORMATION-R2.docx]

SUPPORTING INFORMATION

**Supplementary Table legends**

**Supplementary Table S1.** The lists of RT-qPCR primer sets designed to measure mRNA and enhancer RNA (eRNA) expression of selected genes and binding regions are provided in the first two worksheets. The third worksheet contains the genes and proteins (names and symbols) mentioned in the manuscript.

**Supplementary Table S2.** The genomic coordinates of binding regions belonging to different clusters are provided in the first worksheet. For each region, the occupancy values by RARα and VDR, the label of the binding cluster, the closest annotated gene, the motif scores and the ATAC-seq signal are indicated. The second worksheet contains the number of peaks identified in various data sets.

**Supplementary Table S3.** The first three worksheets contain differentially expressed genes in ligand-stimulated PMA-THP-1 cells. The other worksheets contain lists of various gene sets analyzed in the study.

**Supplementary Table S4.** The first worksheet contains the genomic coordinates of the TSS ± 25 kb of the up-regulated genes, as determined by RNA-seq, along with the RARα and VDR binding regions located within this window. The second worksheet contains RARα and VDR binding regions in TSS ± 25 kb of cooperatively up-regulated genes, that were co-localized with cooperative MED1 (regions where MED1 signals were significantly higher in the combined treatment than in the more effective single treatment (p ≤ 0.05)).

**Supplementary Table S5.** A list of selected studies on pairwise comparisons of nuclear receptor binding regions using ChIP-seq.
